# The E3-ome gene-centric compendium reveals the human E3 ligase landscape

**DOI:** 10.1016/j.cell.2026.01.029

**Published:** 2026-04-02

**Authors:** Ngee Kiat Chua, Tania J. González-Robles, Cameron J. Reddington, Jane Dudley-Fraser, Richard W. Birkinshaw, Jiru Han, Ashleigh Solano, Soon Wei Wong, Tomasz Kochańczyk, Joshua J. Peter, Mark A. Nakasone, Florian Aust, Jacob Munro, Yeh Huei Tong, Julie Iskander, Waruni Abeysekera, Alex Garnham, Hannah Huckstep, Matthew E. Ritchie, Ingrid Wertz, Sarah Hymowitz, Sharad Kumar, Ron C. Conaway, Gilbert G. Privé, Alex N. Bullock, Jeffrey J. Babon, Rachel E. Klevit, Sonja Lorenz, Alessio Ciulli, Eric S. Fischer, Nicolas H. Thomä, Radosław P. Nowak, Brenda A. Schulman, Michael Rapé, Katrin Rittinger, Julia K. Pagan, Melanie Bahlo, Joel P. Mackay, Peter D. Mace, Christopher D. Lima, Ronald T. Hay, David Komander, Bernhard C. Lechtenberg, Claudio A.P. Joazeiro, Michele Pagano, Kay Hofmann, Rebecca Feltham

**Affiliations:** 1 Walter and Eliza Hall Institute of Medical Research, Parkville, Melbourne, VIC 3052, Australia; 2 Department of Medical Biology, University of Melbourne, Parkville, Melbourne, VIC 3010, Australia; 3 Department of Biochemistry and Molecular Pharmacology, New York University Grossman School of Medicine, New York, NY 10016, USA; 4 Laura and Isaac Perlmutter Cancer Center, New York University Grossman School of Medicine, New York, NY 10016, USA; 5 Howard Hughes Medical Institute, New York University Grossman School of Medicine, New York, NY 10016, USA; 6 Department of Precision Medicine, New York University Grossman School of Medicine, New York, NY 10016, USA; 7 Biochemistry Department, School of Biomedical Sciences, University of Otago, Dunedin 9054, New Zealand; 8 Molecular Structure of Cell Signalling Laboratory, the Francis Crick Institute, London NW1 1AT, UK; 9 Robinson Research Institute and Adelaide Medical School, the University of Adelaide, Adelaide, SA 5006, Australia; 10 College of Medicine and Public Health, Flinders Health and Medical Research Institute, Flinders University, Adelaide, SA 5042, Australia; 11 Structural Biology Program, Sloan Kettering Institute, Memorial Sloan Kettering Cancer Center, New York, NY 10065, USA; 12 Centre for Targeted Protein Degradation, School of Life Sciences, University of Dundee, Dundee DD1 5JJ, UK; 13 Research Group ‘Ubiquitin Signaling Specificity’, Max Planck Institute for Multidisciplinary Sciences, 37077 Göttingen, Germany; 14 Lyterian Therapeutics, 630 Gateway Blvd, South San Francisco, CA 94080, USA; 15 The Column Group, 1 Letterman Drive Building D, Suite DM-900, San Francisco, CA 94129, USA; 16 Center for Cancer Biology, University of South Australia, Adelaide, SA 5001, Australia; 17 Adelaide Medical School, the University of Adelaide, Adelaide, SA 5005, Australia; 18 Center for Cancer Biology, College of Health, Adelaide University, Adelaide, SA 5000, Australia; 19 Lyda Hill Department of Bioinformatics, University of Texas Southwestern Medical Center, Dallas, TX 75390-9046, USA; 20 Princess Margaret Cancer Centre, Toronto, ON M5G 1L7, Canada; 21 Department of Medical Biophysics, University of Toronto, Toronto, ON M5G 1L7, Canada; 22 Department of Biochemistry, University of Toronto, Toronto, Ontario M5S 1A8, Canada; 23 Centre for Medicines Discovery, Nuffield Department of Medicine Research Building, University of Oxford, Roosevelt Drive, Oxford OX3 7FZ, UK; 24 Department of Biochemistry, University of Washington, Seattle, WA 98195, USA; 25 Department of Cancer Biology, Dana-Farber Cancer Institute, Boston, MA 02115, USA; 26 Department of Biological Chemistry and Molecular Pharmacology, Harvard Medical School, Boston, MA 02115, USA; 27 Swiss Institute for Experimental Cancer Research (ISREC), École Polytechnique Fédérale de Lausanne, 1015 Lausanne, Switzerland; 28 Institute of Structural Biology, Medical Faculty, University of Bonn, DE-53127 Bonn, Germany; 29 Department of Molecular Machines and Signaling, Max Planck Institute of Biochemistry, D-82152 Martinsried, Germany; 30 Department of Molecular and Cell Biology, University of California at Berkeley, Berkeley, CA 94720, USA; 31 Howard Hughes Medical Institute, University of California at Berkeley, Berkeley, CA 94720, USA; 32 California Institute for Quantitative Biosciences (QB3), University of California at Berkeley, Berkeley, CA 94720-3220, USA; 33 School of Biomedical Sciences, Faculty of Health, Medicine and Behavioural Sciences, The University of Queensland, Brisbane, QLD 4072, Australia; 34 Institute for Molecular Bioscience, The University of Queensland, Brisbane, QLD 4072, Australia; 35 School of Life and Environmental Sciences, University of Sydney, Sydney, NSW 2006, Australia; 36 Howard Hughes Medical Institute, Memorial Sloan Kettering Cancer Center, New York, NY 10065, USA; 37 Division of Molecular, Cellular and Developmental Biology, School of Life Sciences, University of Dundee, Dow Street, Dundee DD1 5EH, UK; 38 Center for Molecular Biology of Heidelberg University (ZMBH), DKFZ-ZMBH-Alliance, 69120 Heidelberg, Germany; 39 Department of Molecular Medicine, the Herbert Wertheim UF Scripps Institute for Biomedical Innovation & Technology, Jupiter, FL 33458, USA; 40 Institute for Genetics, University of Cologne, 50674 Cologne, Germany

## Abstract

To define and systematically characterize the human E3 ubiquitin ligase (E3) landscape, we generated the E3-ome, a compendium of E3s encoded by the human genome. The E3-ome integrates experimental data, bioinformatics, and published research, revealing 672 high-confidence E3s. We standardized E3 classifications to create a unified framework for annotation and comparative analysis. The E3-ome identified several previously unrecognized domains, motifs, E3 candidates, and relationships, expanding the diversity of E3s. Furthermore, the E3-ome mapped the spatial and physiological organization of E3s across human tissues and cell types, revealing context-dependent E3s. Genetic analyses identified disease-associated variants across the E3-ome, linking E3s to diverse human pathologies. Together, these analyses define the human E3 landscape at high resolution and deliver a foundational resource to drive mechanistic and therapeutic discovery.

## Highlights

• Systematic classification defines 672 human E3s across families and classes
• Genetic variants in 26% of E3 genes associate with human phenotypes and disease
• Multi-scale mapping defines E3s across 31 tissues, 38 cell classes, and 17 organelles
• AlphaFold structural comparisons reveal and clarify domain relationships within E3s

## Graphical abstract

**Figure:**
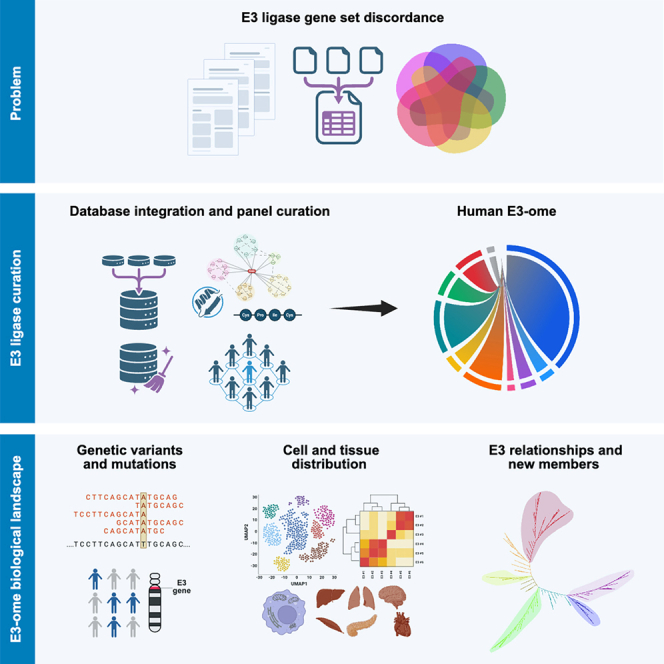

## Teaser

The E3-ome defines the human repertoire of ubiquitin E3 ligases, creating a unified resource that maps their diversity across the ubiquitin and ubiquitin-like systems. By consolidating fragmented knowledge, this framework provides a foundation for studying ubiquitin signaling and accelerating discovery.

## Introduction

E3s, the architects of protein ubiquitination, play a pivotal role in shaping the cellular landscape. Historically, the field has defined an E3 as the terminal enzyme in the ubiquitination cascade that can discharge ubiquitin (Ub) or ubiquitin-like (Ubl) proteins (SUMO-1, SUMO-2, SUMO-3, NEDD8, FAT10, UFM1, ISG15), from an E2 conjugating enzyme (E2) onto a target substrate.^1^^,^^2^^,^^3^^,^^4^^,^^5^^,^^6^ Within the Ub and Ubl system, the specificity of substrate modification is predominantly achieved at the E3 level, which act after the E1 activating and E2 conjugating enzymes, while deubiquitinases (DUBs) reverse ubiquitination by removing Ub from the substrate.^1^^,^^2^^,^^4^^,^^7^^,^^8^ By catalyzing Ub or Ubl transfer to substrates, E3s control protein fate, interactions, and activity, shaping processes such as cell cycle regulation, DNA repair, quality control, and immune responses, contributing to cellular homeostasis and stress adaptation.^9^^,^^10^^,^^11^^,^^12^^,^^13^ The functional repertoire of E3s also includes the attachment of Ub onto non-proteinaceous substrates,^14^^,^^15^ expanding the versatility of E3s in biology.

The E3 families and classes are typically categorized into really interesting new gene (RING), homologous to the E6-AP carboxyl terminus (HECT), RING-between-RING (RBR), and degenerate RING (dRING), such as U-box and SP-RINGs. RING E3s act as scaffolds, directly facilitating Ub transfer from E2s to substrates. Unlike RINGs, HECT E3s form a transient thioester bond with Ub. RBR E3s represent a hybrid mechanism, in which the RING1 domain binds the E2 and transfers Ub to the active site cysteine of the RING2 domain via a transthioesterification reaction (also known as transthiolation), subsequently facilitating the direct modification of the substrate. dRINGs such as the U-box adopt a RING fold despite lacking zinc-ion coordination,^16^ and SP-RINGs are specialized to catalyze SUMOylation of substrates.^17^

E3s operate in diverse configurations, categorized into non-modular and modular forms, ranging from homo- or hetero-oligomeric to multi-subunit complexes. The Cullin RING E3 ligases (CRLs) exemplify complex modular systems, in which multiple protein units work in concert to facilitate substrate modification.^18^ In CRLs, RBX1 typically associates with CUL1/2/3/4A/4B/9 scaffolds whereas the CUL5 scaffold pairs with RBX2. CRLs involve binding of substrates via interchangeable substrate receptors (SRs) defined by specific protein interaction domains (e.g., F-box, BC-box-CUL2-box, BTB, BC-box-CUL5-box) and linking of receptors via adaptors (e.g., SKP1, DDB1, ELOB, ELOC), yielding a variety of CRL configurations. To date, CUL7 has not been shown to be neddylated or to have E3 activity; rather, it acts as a substrate-binding module, although future studies might reveal conditions in which these functions are achieved.^19^

The diversity of E3s underlies their central role in cellular function and disease. For instance, mutations in Parkin are linked to Parkinson’s disease.^20^ In cancer, MDM2 overexpression inhibits the tumor suppressor p53,^21^^,^^22^ and dysfunction of the anaphase-promoting complex/cyclosome (APC/C) is linked to tumor progression.^23^^,^^24^ Although genetic models in mice have clarified disease roles, direct links between human E3s and disease remain limited. Nevertheless, insights from animal models and available human data have enabled drug development strategies, including E3 inhibitors and degraders that hijack E3s to target disease-related proteins.^25^^,^^26^^,^^27^^,^^28^

The E3 landscape is further enriched by Ubl E3s.^3^ SUMO E3s catalyze SUMOylation and influence nuclear processes.^29^ NEDD8 E3s catalyze neddylation, crucial for activating CRLs.^30^ FAT10 E3s mediate FATylation, linked to proteasomal degradation and immune responses.^31^ The UFM1 E3 complex orchestrates UFMylation, key to ribosome recycling and quality control.^32^ ISG15 E3s facilitate ISGylation, critical for immune system function.^33^

Given the complexity and biomedical significance of E3s, an up-to-date and comprehensive resource is needed to accurately curate these enzymes. Existing databases^34^^,^^35^^,^^36^^,^^37^ suffer from incomplete and inconsistent annotation due to variable E3 definitions and rapid field expansion. The E3-ome integrates all currently known human E3s into a unified, standardized reference, using experimental evidence, bioinformatic analyses, and literature curation, providing a comprehensive framework for functional, structural, and biological annotation. To facilitate accessibility, the E3-ome data is available as an online resource on GitHub (https://github.com/FelthamLaboratory/E3-ome) to ensure the resource remains dynamic and adaptable, fostering collaboration and furthering our understanding of Ub/Ubl E3 biology and its implications in disease.

## Results

### Classification of E3s

**Figure 1:**
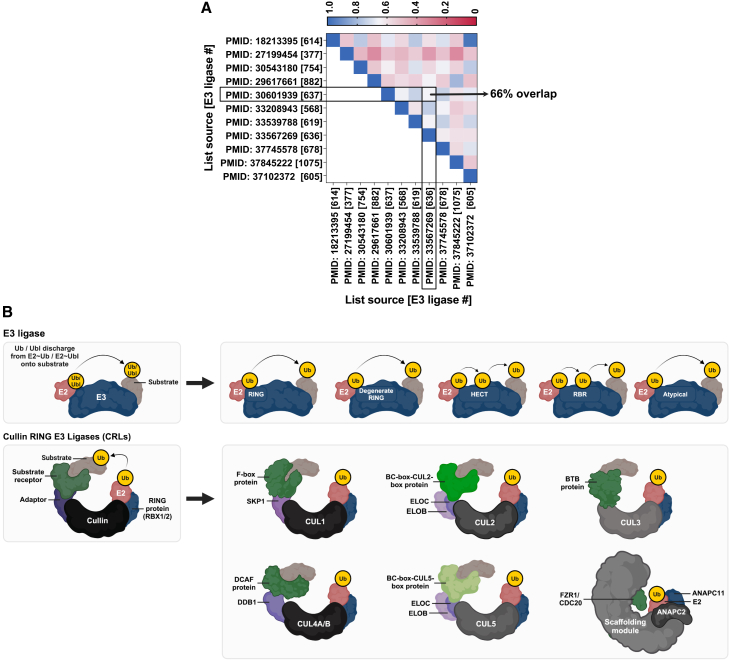
E3 sets across studies and an overview of the E3 landscape (A) Heatmap illustrating the degree of overlap among E3 lists compiled from various studies, identified by their PubMed ID (PMID), with corresponding E3 counts in brackets [###]. Each heatmap cell represents the percentage of E3s common between 2 lists, with darker blue shades indicating higher overlap. (B) E3s (dark blue) transfer ubiquitin (Ub) or ubiquitin-like protein (Ubl) (yellow) from an E2 conjugating enzyme (light red) to target substrates (brown). The landscape includes E3s with specific domains, including RING, degenerate RING, HECT, RBR, and atypical forms vital for target ubiquitination. The diversity of Cullin RING E3 Ligases (CRLs) stems from their varying Cullin scaffolds and the specific SRs (green) employed. See also Figure S1.

A unified E3 classification is essential for standardizing gene sets. Comparison of 11 published lists revealed major inconsistencies, with only 66% gene overlap among lists reporting similar numbers of E3s, underscoring divergent classification methods (Figure 1A). To accurately and comprehensively capture the diversity of E3s, an overarching E3 definition was established. We define an active E3 as a protein that can bind to an E2∼Ub or E2∼Ubl protein intermediate (the tilde [∼] represents a thioester bond between the C-terminus of Ub/Ubl and the active site cysteine within the catalytic UBC domain of the E2) with the ability to catalyze the discharge of Ub or Ubl onto a target substrate or itself (Figure 1B). This definition encompasses modular E3 systems, such as CRLs, in which coordinated activity among multiple protein units enables Ub discharge from the E2∼Ub complex onto substrates (Figure 1B). This definition also includes self-sufficient active E3s encoding both the E3 catalytic domain and the E2 UBC domain (e.g., BIRC6), which can catalyze Ub transfer without an independent E2. In addition, we recognize pseudo-E3s that lack catalytic activity but can play structural or regulatory roles in catalytically active E3 complexes.^38^^,^^39^^,^^40^^,^^41^ For simplicity, we use Ub throughout, unless a specific Ubl is explicitly mentioned.

The E3-ome categorizes E3s based on the presence of specific catalytic domains: RING, dRING, HECT, and RBR (Figure 1B). dRINGs, despite their sequence variations, maintain the structural integrity necessary to, in theory, mediate the transfer of Ub or Ubls to a target substrate. The class of dRINGs encompasses previously classified U-box, SP-RING, U-box-like, RING-like, and RING-variant proteins. Furthermore, we recognize “atypical” E3s as a class including those that do not conform to typical RING, HECT, RBR, and dRING classifications due to distinct structural features, catalytic domains, or mechanism of action (e.g., IR1-M-IR2 region of RANBP2^42^^,^^43^). For CRLs, classification hinges on SRs, which are typically categorized based on specific protein interaction domains or adaptor-binding capacity (e.g., F-box, BC-box-CUL2-box, BTB domain, BC-box-CUL5-box, DDB1-CUL4-associated factors [DCAFs]) (Figure 1B). The interaction domains within SRs generally mediate binding to the Cullin scaffold and/or adaptor proteins, although exceptions can occur on a case-by-case basis (Figure 1B).

### Refining the E3 landscape

**Figure S1:**
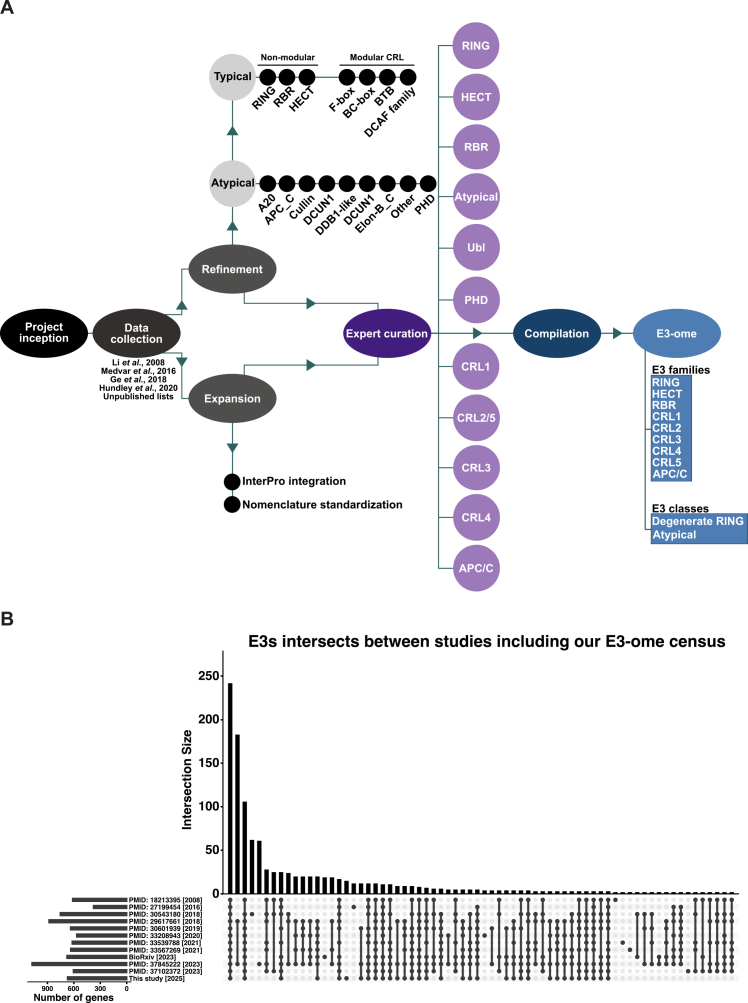
E3 curation workflow and overlaps, related to Figures 1, 2, and 3 (A) The E3-ome project compiled 4 published E3 lists and various unpublished lists. Refinement and expansion followed, separating E3s into typical and atypical groups, incorporating data from the InterPro database, and standardizing nomenclature. E3s were then classified into defined families or classes, with each classification reviewed by curators to ensure accurate and comprehensive classification before being compiled into the E3-ome. (B) The current E3-ome census compared with 11 prior E3 lists to reveal unique and overlapping genes.

To provide a human E3 inventory, 4 lists that categorized E3s were compiled.^37^^,^^44^^,^^45^^,^^46^ We incorporated unpublished lists and literature searches to generate a list of 1,130 human protein-coding genes (Table S1). To expand, standardize, and guide our annotation, InterPro^47^ was used to identify proteins annotated with E3 catalytic domains (RING, U-box, HECT, RBR) and protein interaction domains (F-box, SOCS-box, BTB). Proteins were compared against annotations from both published and unpublished E3 lists to identify overlaps and discrepancies, and to categorize genes into smaller, more specific families or classes. A curation process was established, classifying protein-coding genes into 9 principal E3 families: RING, HECT, RBR, CRL1/2/3/4/5, and APC/C, and 2 classes: dRING or atypical (Figure S1A). This categorization defines the E3-ome and improves dataset clarity and utility. Accurate E3 enumeration focused on 672 high-confidence category 1 genes, whereas 242 category 2 genes were retained pending future evidence (Table S1). This gene-centric approach improves consistency but may underrepresent functional diversity arising from cofactors, complex formation, post-translational modifications, or alternative splicing.

### Curation of RING, dRING, HECT, and RBR E3s

**Figure 2:**
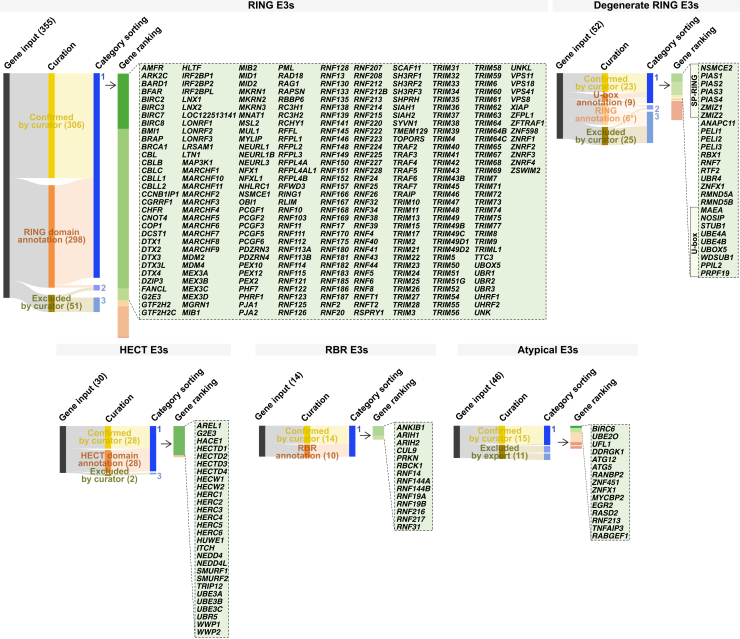
Curation of E3s by catalytic domain and function Sankey diagrams mapping the systematic identification of E3s based on curator input, signature catalytic domains (RING, dRING, HECT, and RBR), or atypical domains. Each diagram represents the workflow from gene input through curation to the categorization into high- and low-confidence groups. High-confidence E3s are highlighted in green call-out boxes. The asterisk (^∗^) denotes that only a subset of dRING inputs were scored for RING presence. dRING of the U-box class was not scored for RING presence but scored for U-box presence. See also Figures S1–S6 and S9.

Non-CRL E3s were categorized based on signature catalytic domain structure and mechanism of action: RING, dRING, HECT, and RBR (Figure 2).

RING E3s represent the largest family and include proteins that function as part of multi-protein complexes, such as the dRING proteins RBX1, RBX2, and ANAPC11, which couple with Cullin scaffolds.^4^^,^^48^^,^^49^ A list of 302 high-confidence RING domain-containing E3s was curated (Figure 2; Table S1B), 285 of which have prior InterPro RING domain annotation (Table S1B). The RING domains are characterized by the presence of 2 zinc ions, typically coordinated by cysteines and histidines arranged in a cross-braced fold.^49^^,^^50^

**Figure S2:**
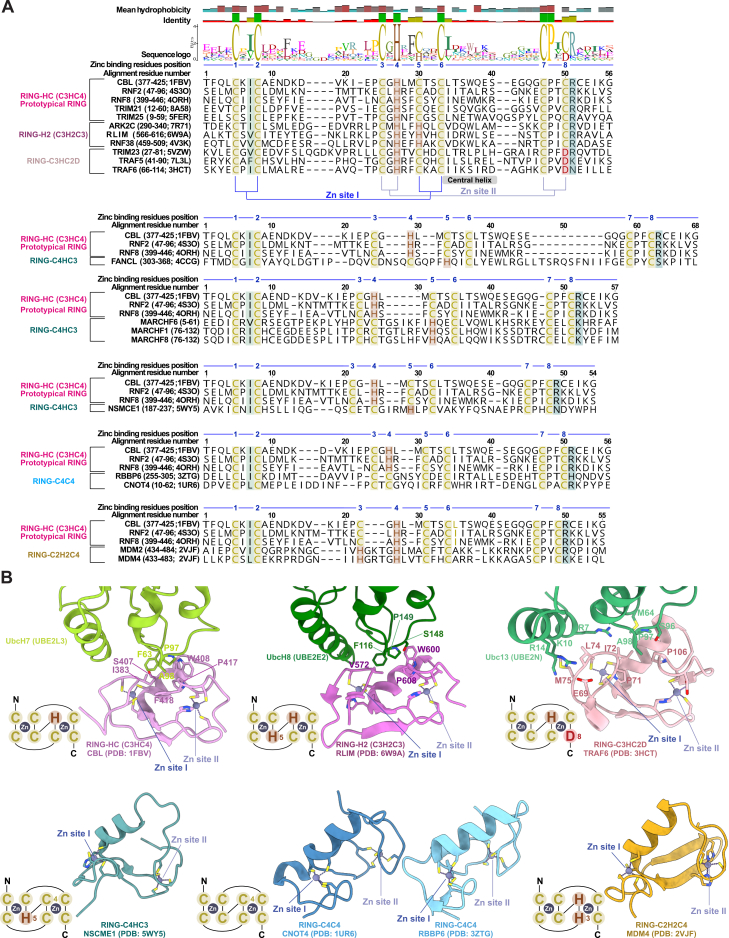
Diversity of the RING protein family, related to Figures 2, 4, and 7 (A) Structure-guided sequence alignment of RING proteins based on the pattern of amino acids used to coordinate zinc. Shown are 6 types of RING domains arrangements: RING-HC (C3HC4), RING-H2 (C3H2C3), RING-C3HC2D, RING-C4HC3, RING-C4C4, and RING-C2H2C4. The hydrophobic residues important for binding of the RING domain to E2 conjugating enzymes are shaded green (before the second cysteine in the zinc coordination set of amino acids). After the eighth zinc-coordinating amino acid generally lies a linchpin residue (shaded teal, with arginine and lysine being the more common amino acids) that is implicated in activating the E2∼Ub conjugate by promoting a “closed” state for Ub discharge. Proteins chosen for the alignment were selected based on availability of experimentally solved structures (PDB identifiers provided), except the MARCHF series of proteins. (B) Structures highlighting the overall conservation of the RING-fold despite the variations in amino acids used in zinc coordination. Key residues involved in the interaction between the RING domain and the respective E2 conjugating enzymes are annotated based on the original findings. For clarity, only a single protomer and a single chain are shown from the experimentally solved structures.

Although the specific arrangement of the zinc-coordinating residues can vary, all RING domains maintain the cross-brace fold (Figure S2). This configuration is theoretically capable of discharging Ub from the E2∼Ub conjugate to transfer Ub onto a substrate, although for many predicted E3s, this functionality has yet to be experimentally confirmed. Furthermore, RING domains vary in their activation states; some are active as monomers (e.g., RNF38, CBL, and CNOT4),^4^^,^^51^ whereas others (e.g., cIAP2, TRAF6, and RNF4) require dimerization.^52^^,^^53^^,^^54^

**Figure S3:**
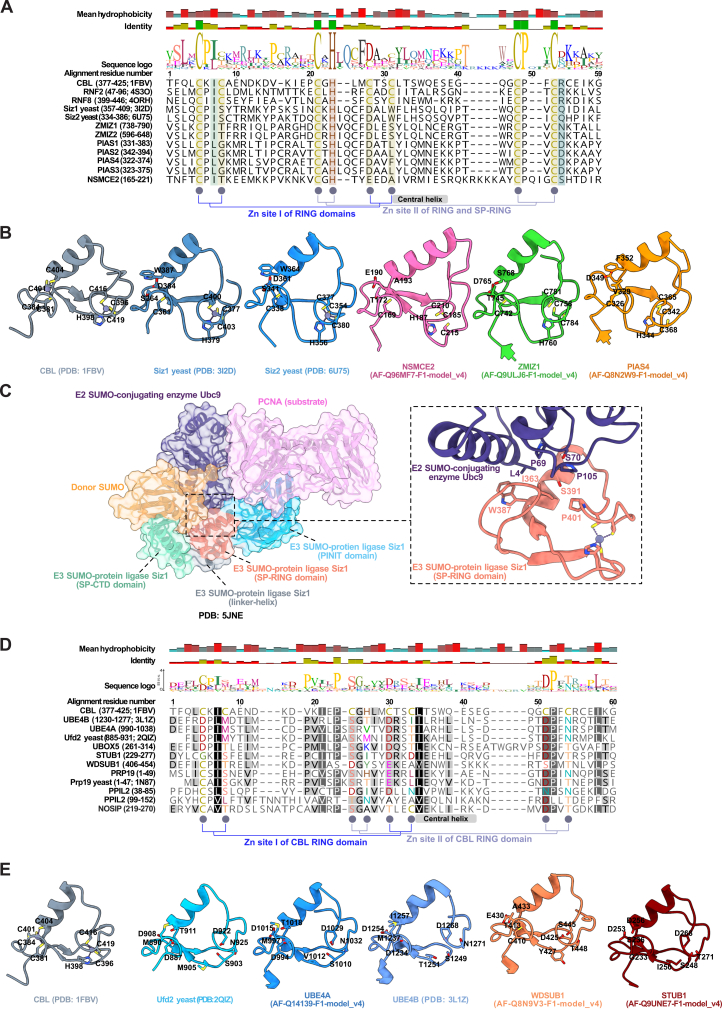
Diversity of the SP-RING and U-box protein classes, related to Figures 2, 4, and 7 (A) Sequence alignment of 3 prototypical RING domains (CBL, RNF2, RNF8) with SP-RING domains from human and yeast. SP-RING domains typically lack the second, fifth, and sixth zinc-coordinating cysteine residues found in RING domains. (B) Structural comparison of the CBL RING domain (a prototypical example) with experimentally solved and predicted structures of SP-RING domains from yeast and human. The overall fold of the CBL RING domain is conserved in SP-RING domains, except SP-RING domains lack the first zinc coordination site. (C) Structure of the yeast Ubc9-donor SUMO/Siz1-backside SUMO/PCNA complex. Backside SUMO is omitted in this depiction. A detailed view of the SP-RING domain interaction with Ubc9 demonstrates a binding mechanism similar to other RING domains with E2s. This interaction involves residues of the central helix and the hydrophobic residue before the second zinc-coordinating cysteine. (D) Sequence alignment of the CBL RING domain with the U-box of several proteins (guided by evidence from curation, literature annotation, and databases). (E) Experimentally solved structures and AlphaFold database structures of regions in (A) to compare the overall fold conservation of the U-box of different proteins with the RING domain of CBL. Residues annotated across all structures are based on pairwise comparison between the CBL RING domain and a potential U-box of interest to identify zinc-coordinating residues that are absent.

The term dRING streamlines non-conventional RING domain-containing proteins with altered zinc coordination and/or structural deviation from the canonical RING cross-brace fold, which may nevertheless retain E3 activity (Figure S3; Table S1C). There were 27 high-confidence dRING proteins, 9 being a class of dRINGs called the U-box proteins and 7 called the SP-RING proteins (Figure 2). SP-RINGs lack residues required to coordinate the first zinc ion yet retain the prototypical RING fold and catalyze SUMO transfer^17^^,^^55^^,^^56^^,^^57^^,^^58^ (Figures S3A–S3C). The U-box lacks zinc-coordinating residues yet adopts the RING fold stabilized via hydrogen bonding and salt bridges, enabling E3 activity^16^^,^^59^^,^^60^^,^^61^ (Figures S3D and S3E). Additional proteins, including RMND5A/5B, UBR4, RTF2, and PELI1/2/3, also feature dRINGs in our classification (Figure 2; Table S1C). The UBR4 hemiRING partially resembles the RING domain and includes an extended β-sheet.^62^ RBX1, RNF7, and ANAPC11 contain 3 zinc ions within their dRINGs.^63^^,^^64^^,^^65^

**Figure S4:**
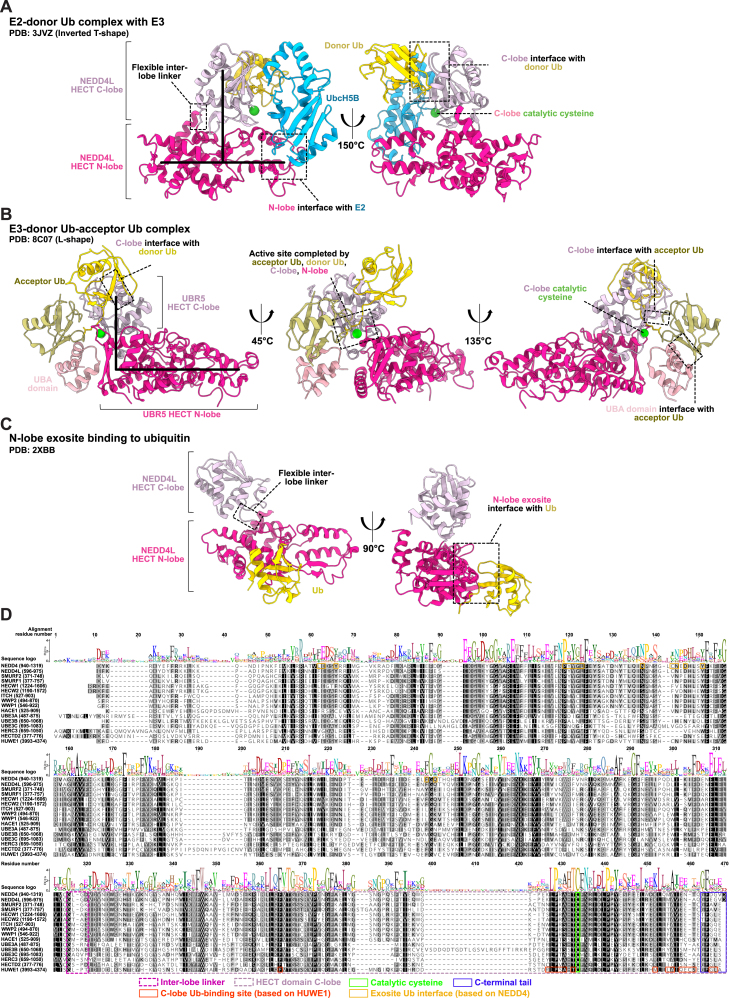
The HECT E3 family, related to Figure 2 (A) Crystal structure of a complex between a UbcH5B∼Ub mimic and the HECT domain of NEDD4L (inactive C922A variant). (B) Detail of a cryo-EM structure of a crosslinked (full-length) UBR5-donor Ub-acceptor complex. This intermediate directs the donor Ub transfer from the E3 to the acceptor. Note that the acceptor orientation toward the HECT domain is stabilized by a UBA domain in UBR5. The acceptor is crosslinked with K48 at the active site, recapitulating K48-linkage formation. (C) Crystal structure of the NEDD4 HECT domain with Ub non-covalently bound at the regulatory exosite on the N-lobe. (D) Sequence alignment of the HECT domain of 16 HECT E3s. The selected sequences represent the 14 most similar to the HECT domain of NEDD4 (except HUWE1, which was included for comparison), based on a FoldSeek search. The inter-lobe linker, C-lobe, C-terminal tail, the donor Ub-binding site, the exosite, and the catalytic cysteine are boxed. The donor Ub-binding site and the exosite were defined by MSDpisa analyses^186^ of HUWE1 (PDB: 6XZ1) and NEDD4 (PDB: 2XBB), selecting residues that experience at least 40% burial upon Ub binding.

28 HECT E3s were curated as high-confidence candidates (Figure 2; Table S1D).^1^^,^^66^^,^^67^^,^^68^^,^^69^ HECT E3s contain the conserved HECT domain comprising 2 lobes, a larger N-terminal lobe and a smaller C-terminal lobe, connected by a short flexible linker (Figure S4). The 2 lobes can dynamically rearrange relative to each other, with the best-characterized, catalytically critical conformations being an inverted T-shape or an L-shape, determined by the positioning of the N-lobe relative to the C-lobe (Figures S4A–S4C). These 2 configurations facilitate distinct steps in the catalytic cycle: Ub discharge from the E2 and Ub transfer from the E3 to a substrate, respectively. The N-lobe contains surfaces that enable contacts with the Ub-charged E2 and an exosite that binds to Ub (Figures S4A–S4C). The C-lobe possesses Ub binding sites, a C-terminal tail that contributes to Ub binding, and the catalytic cysteine residue that forms a thioester bond with Ub (Figures S4A, S4B, and S4D).

**Figure S5:**
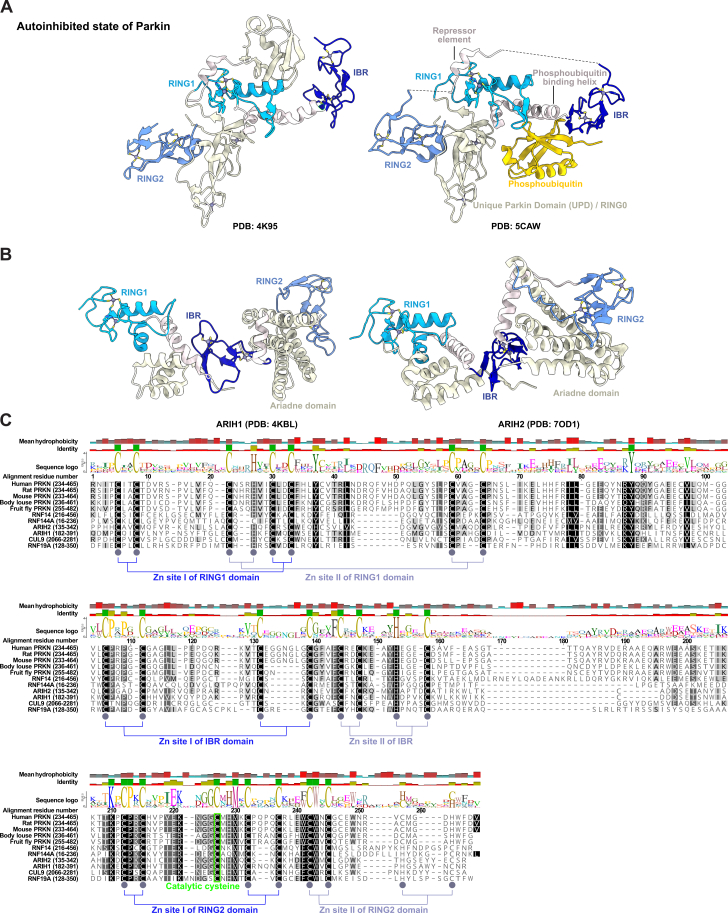
The RBR E3 family, related to Figure 2 (A) Independent structures depicting the autoinhibited state of Parkin (PRKN) with the RING1, IBR, and RING2 regions of the RBR cluster colored in blue. The right structure shows the phosphoubiquitin binding helix, the repressor element, the unique PRKN domain, and phosphoubiquitin. (B) Structure of the RBRs ARIH1 and ARIH2 with the RING1, IBR, and RING2 region of the RBR cluster colored in blue. (C) Sequence alignment of the PRKN RBR region from 5 organisms with 6 other RBR regions from 6 human E3s that are most like PRKN RBR based on FoldSeek.

Curation identified 14 high-confidence E3s as a distinct RBR E3 family (Figure 2; Table S1E), rather than RING E3s as in earlier classifications.^37^^,^^44^^,^^45^^,^^46^ The RBR E3s with their triad of RING1, IBR, and RING2 exhibit more dissimilarities than similarities in structure and mechanism of action compared with the prototypical RING E3s (Figure S5).^70^^,^^71^^,^^72^^,^^73^ The invariably linked RING1-IBR-RING2 triad is required for the RBR scaffolding-catalytic cysteine hybrid mechanism that enables Ub transfer.^70^^,^^74^

### Curation of atypical E3s

A group of E3s that could not be aligned with RING, dRING, HECT, RBR, or CRLs was identified. These were categorized as “atypical” (Figure 2; Table S1F), a term usually reserved for proteins lacking traditional catalytic modules. Some of these atypical E3s were identified through curation of Ubl E3s (Table S1G).

Notable among atypical E3s is MYCBP2, which uses the RING-Cys-Relay (RCR) Ub transfer mechanism, reliant on a catalytic unit comprising a RING domain linked to a tandem cysteine (TC) domain.^75^^,^^76^ ZNF451 SUMO E3 activity requires the SIM and PLRP motifs.^77^^,^^78^ ATG12-ATG5 and UFL1-DDRGK1 complexes exemplify scaffold-type E3s using multi-interface assemblies to form the catalytic module.^79^^,^^80^^,^^81^^,^^82^^,^^83^^,^^84^ BIRC6 and UBE2O are E2/E3 hybrid enzymes, usually referred to as atypical E3s given the E3 domain and E2 UBC domain exist within a single polypeptide chain.^85^^,^^86^^,^^87^^,^^88^^,^^89^

Proteins with PHD domains (e.g., AIRE, JADE2, and DPF2) were considered for classification as atypical E3s due to reports suggesting potential catalytic activities.^90^^,^^91^^,^^92^^,^^93^ Due to the predominant nature of PHD domains in histone mark recognition,^94^^,^^95^^,^^96^ it was not possible to confidently categorize all PHD domain-containing proteins as E3s, nor can E3 activity be assumed for all PHD domains. Conflicting evidence surrounds AIRE functioning as an E3 via its PHD domain.^97^^,^^98^ For DPF2, cell-based systems suggested E3 activity for the PHD domain, but *in vitro* biochemical evidence is absent.^93^ To date, only the PHD domain of PHF7 has been shown to be involved in E2 recruitment, along with the RING domain, with productive *in vitro* E3 activity requiring the RING, PHD, and extended PHD domains.^99^

**Figure S6:**
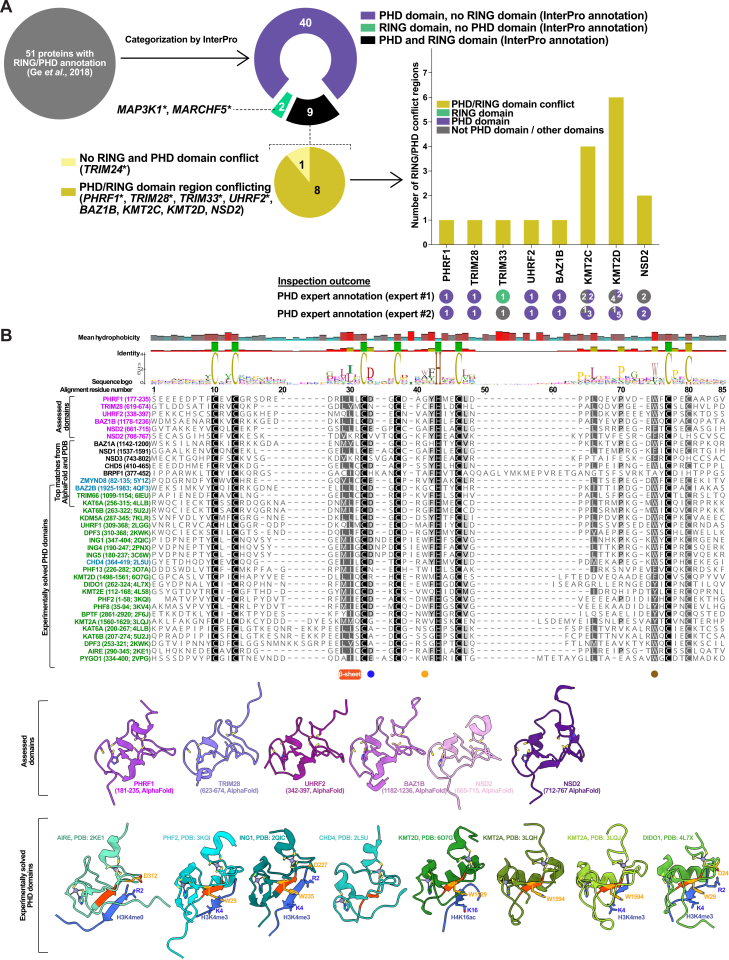
Resolving RING/PHD domain conflicts, related to Figure 2 (A) The RING/PHD domain family of E3s from Ge et al. (2018)^45^ were initially annotated for the presence of PHD and RING using InterPro. Proteins with both RING and PHD domain annotations were analyzed for conflicting annotations on InterPro. Conflict regions were further analyzed by PHD domain curators to identify PHD domains. Proteins with an asterisk (^∗^) are high-confidence RING E3s. (B) Multiple sequence alignment showing residues assessed for RING/PHD domain conflicts from PHRF1, TRIM28, UHRF2, BAZ1B, and NSD2 (purple) compared with experimentally solved PHD domains (green and blue). The most similar domains (from AlphaFold and PDB) compared with the queried domains of PHRF1, TRIM28, UHRF2, BAZ1B, and NSD2 are also indicated. Proteins with experimentally solved structures (green and blue) have PDB identifiers indicated. A key β-sheet region of some PHD domains interacting with histones is shown (orange bar). Alignment also shows amino acid positions in which some PHD domains could use a tryptophan (orange circle) or an aspartate (blue circle) to bind histones. An aromatic acid, typically tryptophan, is also usually found in PHD fingers positioned before the last cysteine pair (gold circle). (C) AlphaFold database predicted structures from the RING/PHD conflicting regions of PHRF1, TRIM28, UHRF2, BAZ1B, and NSD2 compared with experimentally solved PHD domains.

PHD domain annotation requires caution, as exemplified by MEKK1, which was initially misclassified to contain a RING domain.^100^^,^^101^^,^^102^ The curation identified one study that classified 51 proteins as RING/PHD (Table S1H).^45^ Integration of experimentally solved PHD structures bound to histone partners enabled discrimination between true RING and PHD domains (Figure S6).^95^^,^^103^^,^^104^^,^^105^^,^^106^^,^^107^ Some notable mentions are TRIM24, which contains both a PHD domain (residues 828–871) and a RING domain (residues 56–131), and TRIM33, which had conflicting PHD and RING annotations (residues 124–185), but was confirmed to have only a RING domain.

Proteins with diverse domains reported to have E3 activity were also identified (Table S1F). ZFP91 E3 activity is suggested to be mediated by the second basic zinc finger,^108^ TRIM16 and TRIM29 E3 activity through the B-box,^109^^,^^110^ RNF213 E3 activity through the RZ finger,^15^ and RABGEF1 E3 activity through the A20-type zinc finger.^111^^,^^112^ It is unlikely, though, that all proteins with zinc fingers exhibit E3 activity. For instance, the B-box zinc finger in CYLD was tested against 16 different E2s and failed to demonstrate any *in vitro* E3 activity, leading to its exclusion as a catalytic E3 domain.^113^ Out of the 46 putative atypical E3s, 15 were curated as high-confidence (Figure 2; Table S1F).

### Curation of the human CRL SRs

Given their role in CRL substrate specificity, non-catalytic SRs were included in the curation, as excluding them would underestimate E3 functional breadth. Analysis of published and unpublished E3 lists identified 83 putative CRL1 SRs, 87 for CRL2/CRL5, 195 for CRL3, 263 for CRL4, and 2 for APC/C. Following curation, a set of high-confidence CRL SRs was identified: 73 for CRL1, 32 for CRL2, 97 for CRL3, 49 for CRL4, 53 for CRL5, and 2 for APC/C (Tables S1I–S1M).

**Figure 3:**
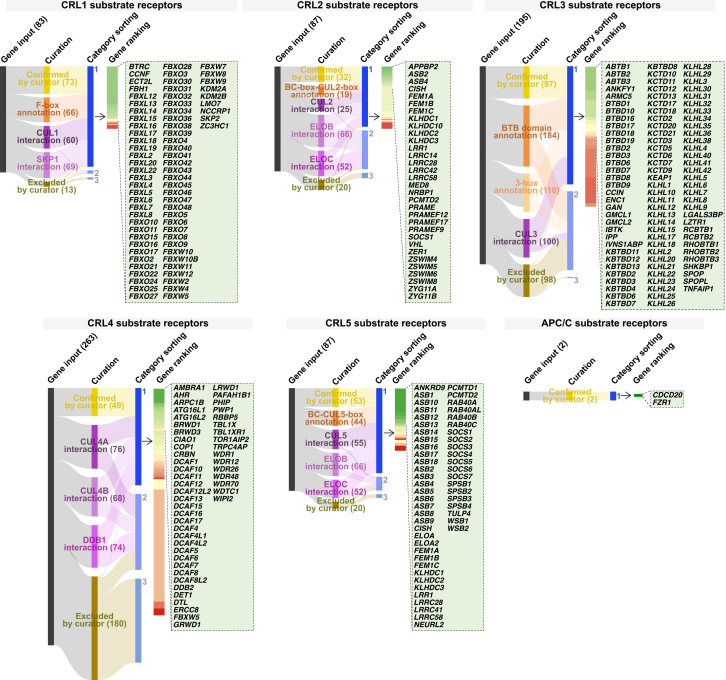
Curation of CRL SRs to enumerate CRL diversity Sankey diagrams mapping the systematic identification of CRL SRs based on curator input, database annotations, and available interaction data. Each diagram depicts the workflow from initial gene input through curation to the categorization into high- and low-confidence groups. High-confidence SRs are highlighted in green call-out boxes. See also Figures S1 and S7–S9.

**Figure S7:**
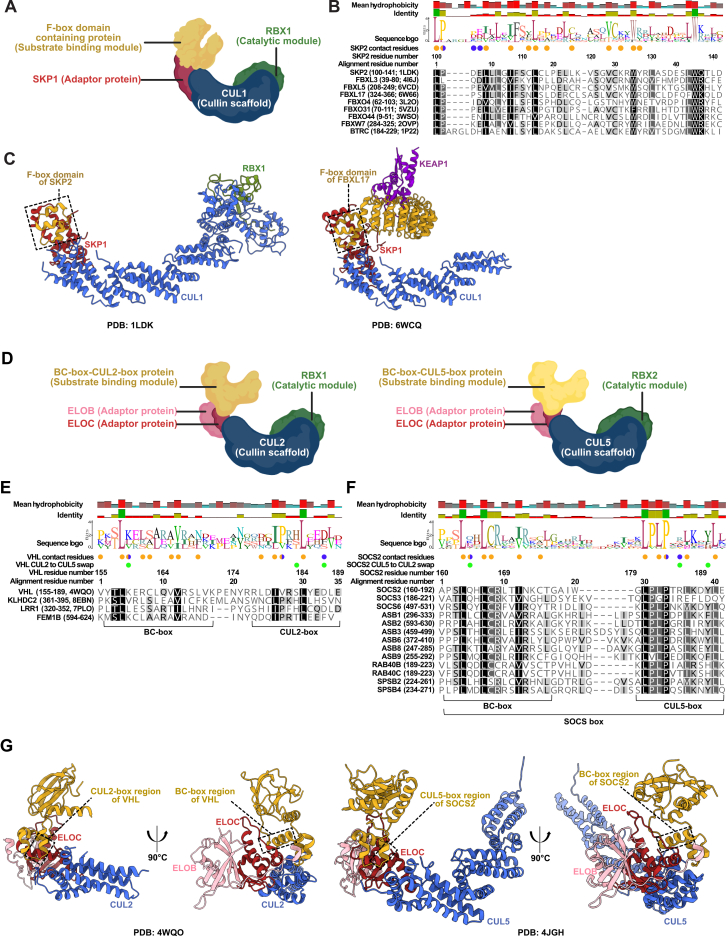
Diversity of CRL1, CRL2 and CRL5, related to Figure 3 (A) Schematic representation of the CRL1 complex highlighting the substrate receptor (F-box containing protein), the adaptor protein (SKP1), Cullin scaffold (CUL1), and catalytic module (RBX1). (B) Structure-guided sequence alignment of the F-box from 9 proteins using Clustal Omega. Proteins shown in the alignment were selected based on the availability of experimentally solved structures showing CRL1 complex formation. The residues of SKP2 that contact CUL1 (blue circles) and SKP1 (orange circles) are annotated based on findings from prior studies.^63^^,^^187^ (C) CRL1 structures showing the F-box (boxed) interacting with SKP1 and CUL1. (D) Schematic representation of CRL2 and CRL5 complexes. (E) Sequence alignment of known SRs of the CRL2 complex. Proteins were selected based on an alignment generated previously.^188^ The alignment highlights the BC-box region and the CUL2-box region. The residues of VHL that contact CUL2 (blue circles) and ELOC (orange circles) are annotated based on prior findings.^188^^,^^189^^,^^190^ Residues of VHL that, when mutated, change the affinity of VHL from CUL2 to CUL5 are also indicated (green circles) based on prior experiments.^190^ (F) Sequence alignment of high-confidence CRL5 SRs using Clustal Omega. The alignment highlights the BC-box region and the CUL5-box region. The residues of SOCS2 that contact CUL5 (blue circle) and ELOC (orange circle) are indicated based on prior findings.^190^^,^^191^^,^^192^ Residues of SOCS2 that, when mutated, change the affinity of SOCS2 from CUL5 to CUL2 are also indicated (green) based on prior experiments.^190^ Proteins chosen for this alignment have at least 3 independent studies reporting high-throughput interactions with CUL2 and at least 3 independent studies showing interactions with ELOB and ELOC (considering both high-throughput and low-throughput studies). Alignments were generated with guidance from past studies.^193^^,^^194^^,^^195^ (G) Left, structure of the CRL2^VHL^ complex highlighting the binding of VHL to CUL2 and ELOC through the CUL2-box and BC-box. Right, structure of the CRL5^SOCS2^ complex, highlighting the binding of SOCS2 to CUL5 and ELOC through the CUL5-box and BC-box.

Of the 83 considered CRL1 SRs, 66 proteins had an InterPro F-box annotation, CUL1 interaction was reported for 60 proteins, and SKP1 interaction was reported for 69 proteins. Overall, 73 proteins were classified as high-confidence CRL1 SRs (Figure 3; Table S1I). Notable high-confidence receptors included FBXL3, FBXL5, FBXO31, and SKP2, all of which exhibit structural evidence for binding to SKP1 and CUL1 (Figures S7A–S7C).

CRL2 SRs are usually characterized by their ability to bind to CUL2, ELOB, and ELOC, enabled by the BC-box-CUL2-box (Figures S7D, S7E, and S7G). The related CRL5 SRs are usually characterized by binding to CUL5, ELOB, and ELOC through the BC-box-CUL5-box (also known as SOCS-box) (Figures S7D, S7F, and S7G). Annotations of the CUL2-box (ϕPxxϕxxxϕ) and CUL5-box (ϕxxLPϕPxxϕxxY/FL) are available from prior literature.^114^^,^^115^^,^^116^ The InterPro database contains only the SOCS-box annotation, but BC-box-CUL2-box annotations are absent. Amongst our 32 high-confidence CRL2 SRs, 23 had evidence of CUL2 interaction, 27 had evidence of ELOB interaction, 18 had evidence of ELOC interaction, and 19 had prior literature annotation of a BC-box-CUL2-box (Figure 3; Table S1J). Of the 53 high-confidence CRL5 SRs, 50 had evidence of CUL5 interaction, 50 had evidence of ELOB interaction, 42 had evidence of ELOC interaction, and 44 had prior annotation of a SOCS-box (Figure 3; Table S1J). Although the Cullin pairing for several BC-box proteins remains to be clearly established, APPBP2, KLHDC2, VHL, and ZYG11B were some of the highest-ranked CRL2 SRs, whereas ASB9, SOCS2, and SPSB4 were some of the highest-ranked CRL5 SRs.

**Figure S8:**
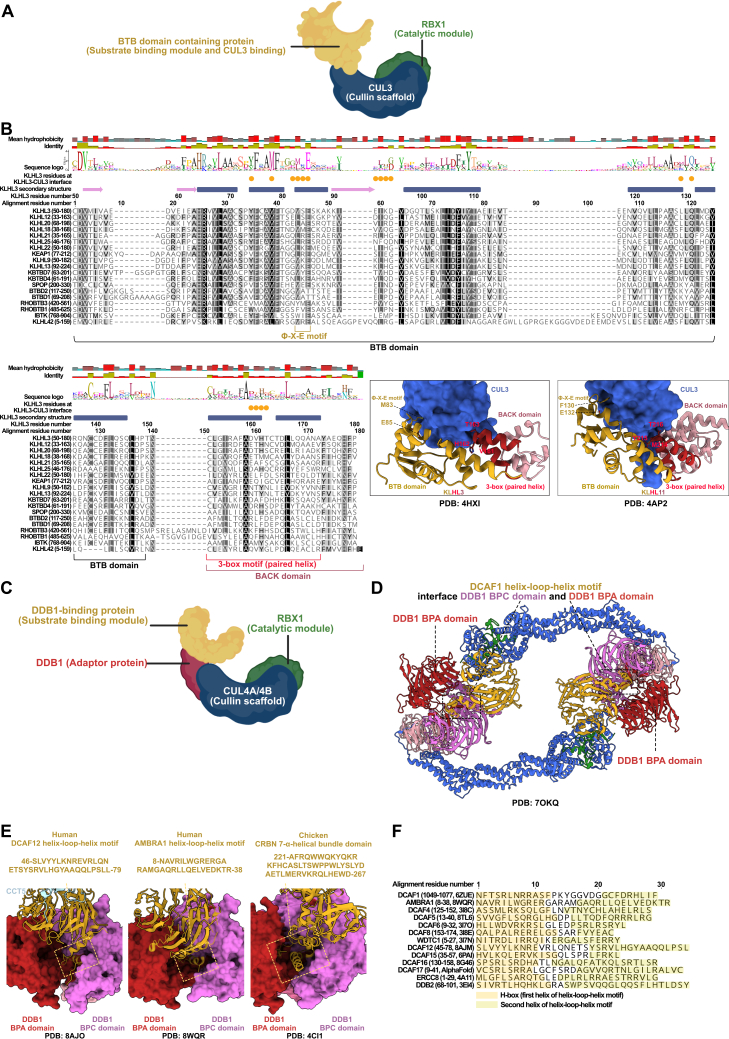
Diversity of CRL3 and CRL4, related to Figure 3 (A) Schematic representation of the CRL3 complex, highlighting the substrate receptor (BTB domain-containing protein), Cullin scaffold (CUL3), and catalytic module (RBX1). (B) Sequence alignment of the BTB domain from a subfamily of BTB proteins that possess a BACK domain. Proteins chosen for this alignment have at least 3 high-throughput and 3 low-throughput independent studies showing interaction with CUL3. The 2 structures are shown to highlight key residues of the φ-X-E motif (within the BTB domain) and the 3-box motif (within the BACK domain) from KLHL3 and KLHL11 contributing to interactions with CUL3. The residues of KLHL3 that contact CUL3 (orange circle) are indicated based on findings from.^196^ The 3-box motif forms the first 2 helices (paired helix) of the BACK domain. (C) Schematic representation of the CRL4 complex, highlighting the substrate receptor (DDB1-binding protein), the adaptor protein (DDB1), Cullin scaffold (CUL4A/CUL4B), and catalytic module (RBX1). (D) The structure represents a dimeric CRL4^DCAF1^ E3 complex. The interchangeable substrate receptor DCAF1 is shown bound to DDB1 through a helix-loop-helix motif. The 3 β-propeller domains of DDB1 are indicated as DDB1 BPA, DDB1 BPB, and DDB1 BPC. (E) Structures depicting the helix-loop-helix motif of CRL4 SRs (DCAF12 and AMBRA1) bound to DDB1 through contacts made in the BPA and BPC cleft of DDB1. CRBN is distinct from other CRL4 SRs in that it does not utilize a helix-loop-helix motif but instead a 7-helical bundle to bind to both BPA and BPC. (F) Sequences of helix-loop-helix motif DDB1-binding proteins. The 2 distinct helices of the helix-loop-helix motif are shaded in 2 shades of yellow (left H-box, right second helix). Regions were highlighted based on experimentally solved structures and AlphaFold structures.

The CRL3 family typically relies on SRs containing a BTB domain, but not all BTB-domain-containing proteins can fulfill this role.^117^^,^^118^^,^^119^^,^^120^^,^^121^ We generalize that BTB domain-containing proteins possessing a Φ-X-E motif (within the BTB domain) and 3-box motif (within the BACK domain) can be CRL3 SRs as typically found in the KLHL family of proteins (Figures S8A and S8B).^118^^,^^120^^,^^121^^,^^122^ In addition, many, but not all, of the BTB-containing KCTD proteins are CRL3 SRs, despite the absence of the Φ-X-E and 3-box motifs. Instead, these proteins bind CUL3 through their BTB domains and engage other surfaces on adjacent BTB subunits in oligomeric assemblies.^123^^,^^124^^,^^125^^,^^126^ Of the 195 potential CRL3 SRs, there were 97 high-confidence candidates based on the presence of the 3-box motif and/or interaction evidence with CUL3 (Figure 3; Table S1K).

The CRL4 family initially included 263 potential CRL4 SRs. After evaluation, the high-confidence set comprises 49 proteins (Figure 3; Table S1L). CRL4 SRs characteristically bind the DDB1 adaptor through a helix-loop-helix motif, which, despite showing very low sequence similarity, maintains structural similarity (Figures S8C–S8F). Earlier findings suggested that WDXR motifs within WD40 repeats facilitated DDB1 binding, but this motif is not universally present across all CRL4 SRs, and newer discoveries reveal a lack of direct DDB1 interaction.^127^^,^^128^ The helix-loop-helix motif, on the other hand, is buried within the β-propeller C and β-propeller A clefts of DDB1, dictating interaction specificity.^128^^,^^129^^,^^130^^,^^131^ Importantly, the binding mode of CRL4 SRs to DDB1 can be degenerate, such as in CRBN, which contains a 7-α-helical bundle to engage with DDB1.^132^

The APC/C is also a multi-subunit CRL. For the APC/C, ANAPC11 is the RING protein contributing catalytic activity, ANAPC2 is the Cullin-related subunit, and there are only 2 known SRs, CDC20 and FZR1 (Figure 3; Table S1M).^133^^,^^134^ CUL9 is the only known example of a chimeric CRL/RBR complex, harboring both the Cullin domain and the RBR domain within the single polypeptide to enable E3-E3 activity.^135^

### Uncovering uncharacterized E3s and motifs

**Figure 4:**
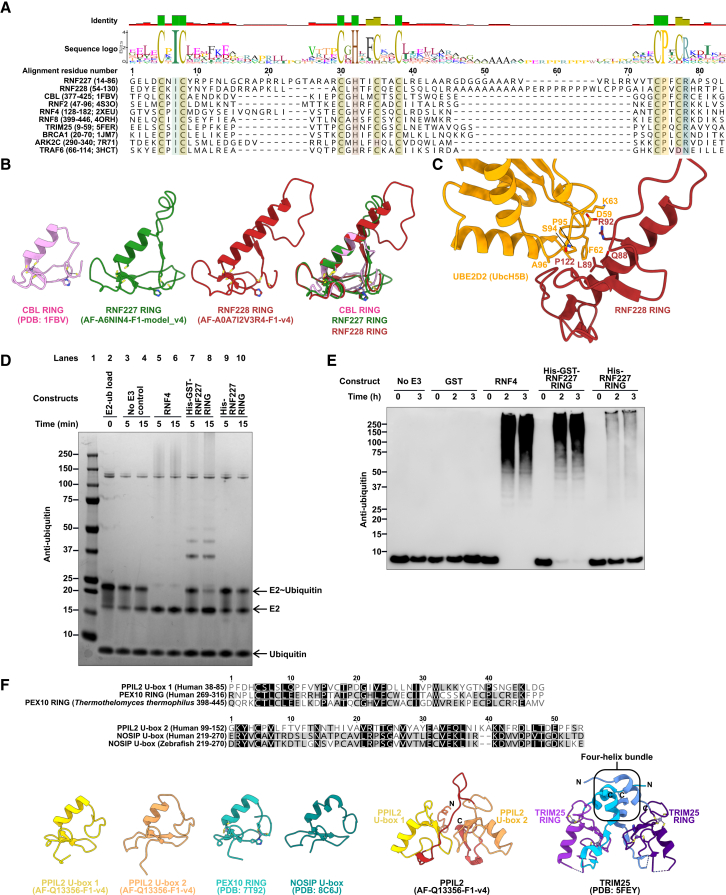
New proteins and motifs of the E3-ome (A) Sequence alignment of the RING domains of RNF227 and RNF228 with the RING domains from other experimentally validated E3s (PDB IDs provided). (B) Structural comparison of the RNF227 and RNF228 RING domains with CBL. (C) Structural prediction of RNF228 interacting with UBE2D2. (D) E2∼Ub discharge assay with the RING domain of RNF227 and full-length RNF4. Experiment is representative of 3 independent experiments. (E) *In vitro* auto-ubiquitination assay with the RING domain of RNF227 and full-length RNF4. Experiment is representative of 3 independent experiments. (F) PPIL2 U-box 1 and U-box 2 comparison with the structurally similar PEX10 RING domain, NOSIP U-box, and TRIM25 dimer. See also Figure S9.

Curation identified potential genes included only in the E3-ome census (e.g., *RNF227*, *RNF228*, *PRAMEF12, KLHDC1, ZNFX1*), with some already shown to be functional CRL SRs or active E3 enzymes.^115^^,^^136^^,^^137^ Additions to the RING family include RNF227 and RNF228. Both have the key C3HC4 zinc coordination and RING fold like that of CBL (Figures 4A and 4B), with UBE2D2 predicted to bind to the RNF228 RING (Figure 4C).

**Figure S9:**
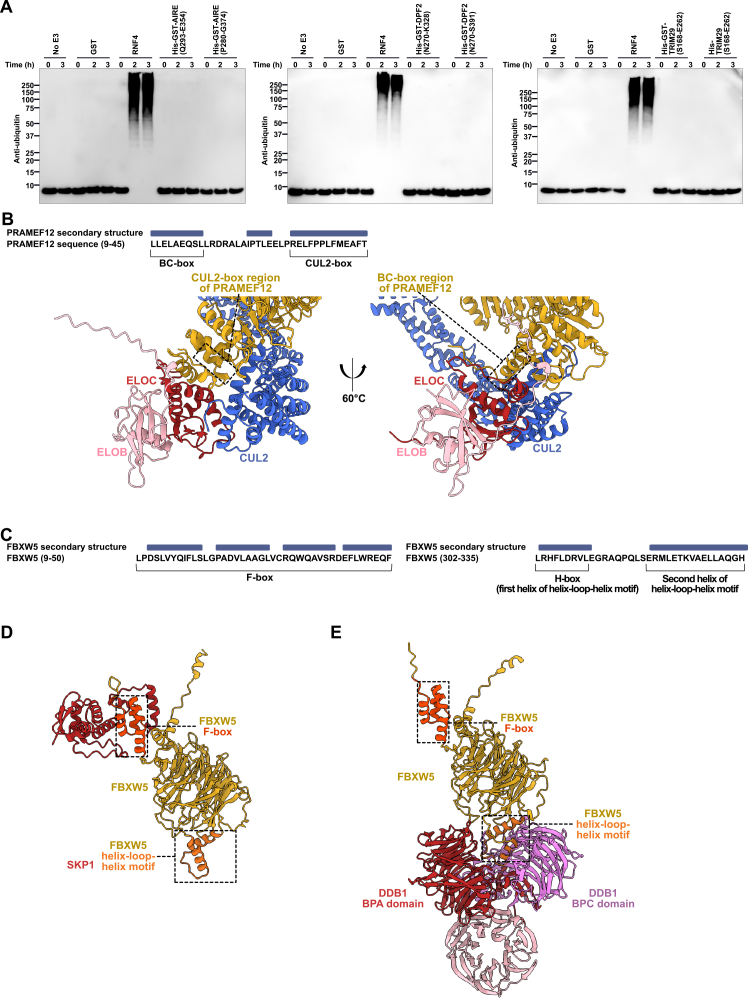
E3 activity tests and CRL substrate receptor motifs, related to Figures 3 and 4 (A) PHD domains (from AIRE and DPF2) and B-box (from TRIM29) were tested for auto-ubiquitination activity. Experiments are representative of 3 independent experiments. (B) PRAMEF12 features a BC-box and CUL2-box with predictions of binding to CUL2, ELOB, and ELOC. (C) Annotation of the FBXW5 F-box and helix-loop-helix motif. (D and E) Predictions of FBXW5 F-box binding to SKP1 and helix-loop-helix motif binding to DDB1 by AlphaFold3.

RING and dRING E3s typically function as scaffolds that position the E2∼Ub conjugate for Ub transfer. RNF227’s intrinsic E3 activity was assessed using E2 discharge assays with recombinant E1, E2, and E3 proteins, monitoring E2∼Ub intermediate depletion alongside polyubiquitin formation assays measuring auto-ubiquitination of GST-RNF227 under established conditions.^54^^,^^138^^,^^139^^,^^140^ These assays confirmed the RNF227 RING could discharge Ub from the E2∼Ub conjugate (Figure 4D) and produce polyubiquitin (Figure 4E). Unlike the RNF227 RING, PHD domains (from DPF2 and AIRE) and the B-box (from TRIM29) could not produce polyubiquitin chains in the same experimental setup (Figure S9A).

We also identified motifs not reported previously. PPIL2 contains a tandem U-box arrangement like that of the active TRIM25 RING dimer (Figure 4F), and an uncharacterized predicted BC-box and CUL2-box in PRAMEF12 (residues 9–45). Through these motifs, PRAMEF12 is predicted to bind to CUL2, ELOB, and ELOC (Figure S9B). We also identified a cryptic helix-loop-helix motif in FBXW5 alongside a known F-box (Figures S9C–S9E). Structural predictions showed that the helix-loop-helix motif could bind DDB1, and the F-box binds to SKP1 (Figures S9C–S9E), suggesting possible simultaneous formation of CRL1 and CRL4 complexes.

### Integrative analysis of the human E3-ome across tissues, cells, and disease

To address gaps in understanding E3 roles in human biology, we used the E3-ome to integrate transcriptomic, phenotypic, cancer gene, and localization datasets.

**Figure 5:**
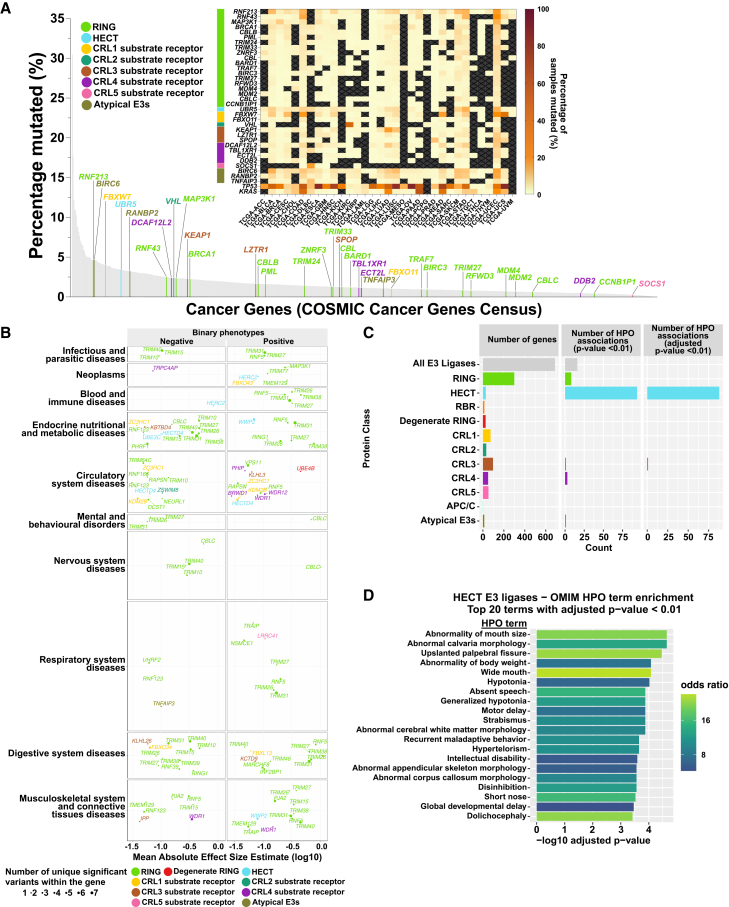
Integration of E3-ome genes with human genetic variant and mutation data (A) Intersection of genes from the E3-ome with the Cancer Genes Census in the Genomics Data Commons portal. (B) E3-ome genetic variants significantly associated (*p* ≤ 1e–8) with human phenotypes as identified in the AZPheWAS Portal. (C) Associations of the E3-ome genes with human phenotypes through Human Phenotype Ontology (HPO) analyses (Fisher's exact test). (D) HECT E3 associations with Online Mendelian Inheritance in Man (OMIM) phenotype terms (Fisher's exact test). See also Figure S10.

Examining cancer genes via the public Genomic Data Commons portal, 34 E3-ome genes were classified as cancer genes, covering 9,871 mutations, including established cancer genes such as *BRCA1* and *KEAP1* (Figure 5A; Table S1N). Clinically observed mutations in these genes can compromise E3 functional integrity.^38^^,^^141^^,^^142^^,^^143^^,^^144^^,^^145^ *RNF213* is comparatively an understudied cancer gene but is the most frequently mutated E3 in the E3-ome, featuring 1,050 mutations across 781 cancer patients. The RNF213 RING mutation C4017Y likely disrupts the RING fold, whereas a *de novo* variant at the same position (C4017S), linked to Moyamoya disease, reduces RING domain activity.^146^^,^^147^ Intriguingly, 3 mutations within the RNF213 E3 module (A3927T, D4013N, S4118F) are common to both cancer and Moyamoya disease patients, highlighting a potentially shared molecular mechanism that connects distinct pathologies.^148^

**Figure S10:**
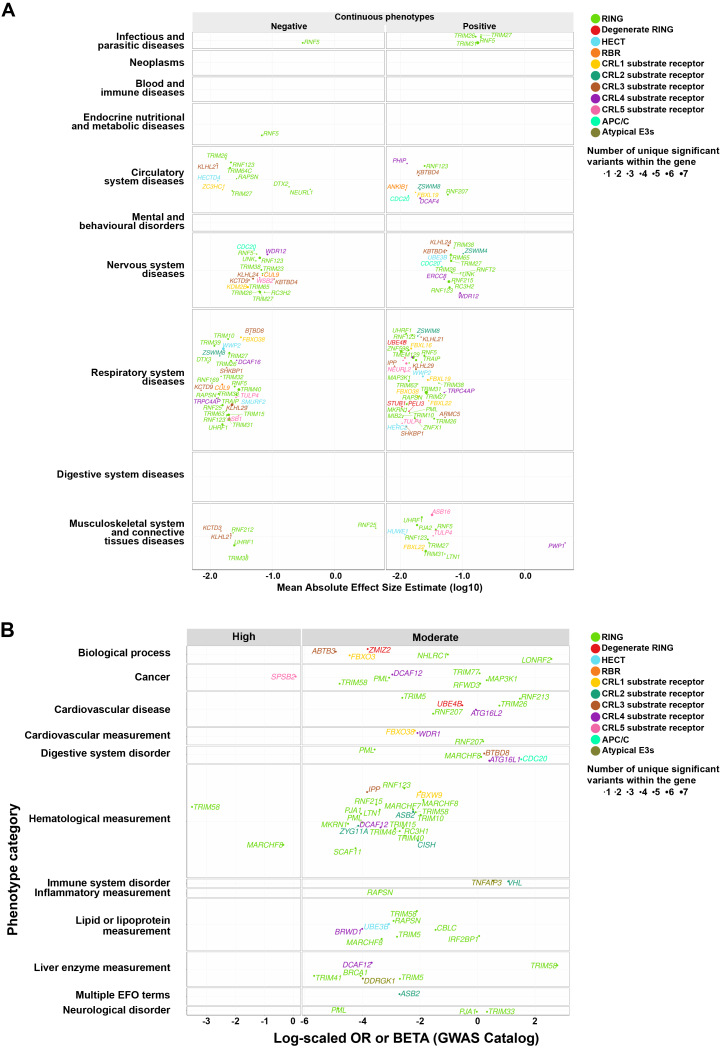
Gene-phenotype associations of E3s, related to Figure 5 (A) Genes from the E3-ome curation that contain genetic variants significantly associated (*p* ≤ 1e–8) with human continuous phenotypes as identified in the AZPheWAS portal. (B) Genes from the E3-ome curation that contain genetic variants significantly associated with human phenotypes (*p* ≤ 5e–8), as reported in the GWAS catalog.

The broader impact of E3s on human phenotypes was examined using the AstraZeneca PheWAS (AZPheWAS) Portal,^149^ which includes gene-phenotype associations from ∼420,000 individuals of European ancestry (Figures 5B and S10A). We identified 224 significant genetic variants across 107 genes with diverse phenotypic effects. For example, a synonymous variant rs1005902 (c.6340A>C) in *HECTD4* is associated with reduced risk of hypothyroidism and hypertension, whereas a synonymous variant rs1062070 (c.471A>G) in *RNF5* is linked to increased risk of diabetes, malabsorption, anemia, and systemic lupus erythematosus (Figure 5B; Table S1O). We next examined phenotypes derived from Online Mendelian Inheritance in Man (OMIM) via the human phenotype ontology (HPO),^150^ and from the genome-wide association study (GWAS) Catalog.^151^ HECT E3s were enriched for Mendelian disorders (Figures 5C and 5D; Table S1P), with 12 of 28 HECTs linked to a spectrum of human phenotypes, including global developmental delay, absent speech, and motor dysfunction, which are features of neurodevelopmental disorders. For instance, *UBE3A* is implicated in Angelman syndrome, whereas *HERC2* is implicated in Prader-Willi syndrome (Table S1P). From the GWAS Catalog, we identified 8,222 variants located within or near E3-ome genes that are associated with human phenotypes (Figure S10B; Table S1Q). One example is the *MARCHF8* missense variant rs7908745 (c.796T>C; p.Tyr266His), which was significantly associated with red blood cell and lipid-related traits.

**Figure 6:**
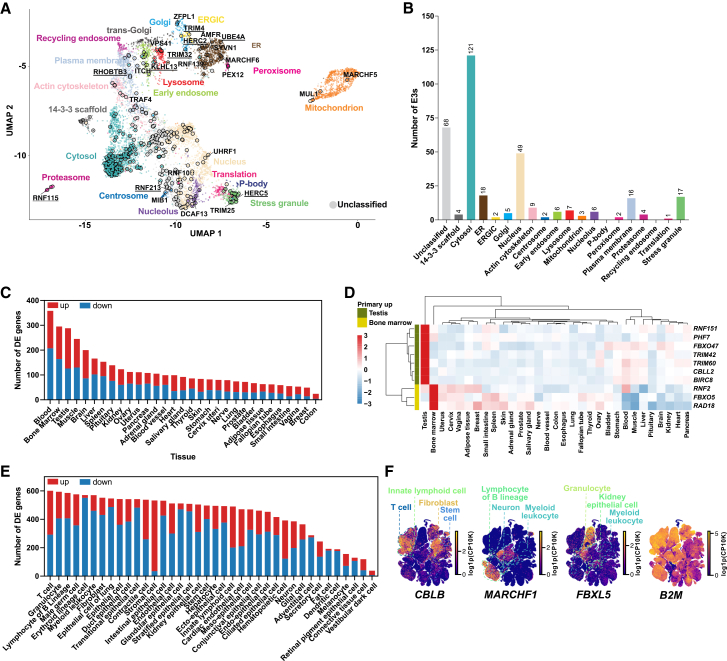
Human E3-ome distribution by cellular localization and gene expression (A) Human E3-ome proteins detected within the HEK293T proteome are indicated by circles. Black text denotes proteins with independent experimental evidence supporting their cellular localization. Proteins identified by organelle profiling^152^ are underlined. (B) Enumeration of E3-ome distribution in HEK293T proteome based on an organelle profiling study.^152^ (C) Number of differentially expressed genes across 31 GTEx human tissues, defined by FDR < 0.05 and log2 fold-change ≥ 1. (D) Heatmap reflects some of the top E3-ome genes that are significantly upregulated primarily in one tissue. Values represent row-wise Z-scores of log2 fold-changes across tissues (for visualization) with red indicating relative upregulation and blue indicating relative downregulation. (E) Number of differentially expressed genes across 38 broad cell classes within Tabula Sapiens, defined by adjusted *p*-value < 0.05. (F) Examples of E3-ome genes that show uniquely upregulated expression in specific classes of cells. *B2M* is included to depict broad expression. Feature plots show single-cell expression projected onto a precomputed Tabula Sapiens UMAP. Dashed outlines highlight regions corresponding to the indicated cell classes. Expression values are calculated from decontX-corrected count as log1p(CP10K) (per-cell counts normalized to 10,000 total counts, followed by log1p transformation). See also Figure S11.

To explore cellular E3 distribution, we integrated the human E3-ome with the most extensive proteome-wide organelle profiling in HEK293T cells.^152^ 272 E3-ome proteins were mapped across 17 cellular compartments, yielding confident localization assignments for 40% of the E3-ome (Figure 6A; Table S1R). This included known organelle-resident E3s in the mitochondria (MARCHF5, MUL1), endoplasmic reticulum (AMFR, SYVN1), and nucleus (UHRF1). E3s were most abundant in the cytosol, nucleus, ER, plasma membrane, and stress granule (Figures 6A and 6B). To our knowledge, this organelle profiling dataset is not yet integrated into UniProt localization annotations. The organelle profiling also revealed unreported localizations, such as RNF213 (centrosome), UBE4A (ER), TRIM4 (ERGIC), RNF115 (proteasome), and HERC5 (stress granules) (Figure 6A).

**Figure S11:**
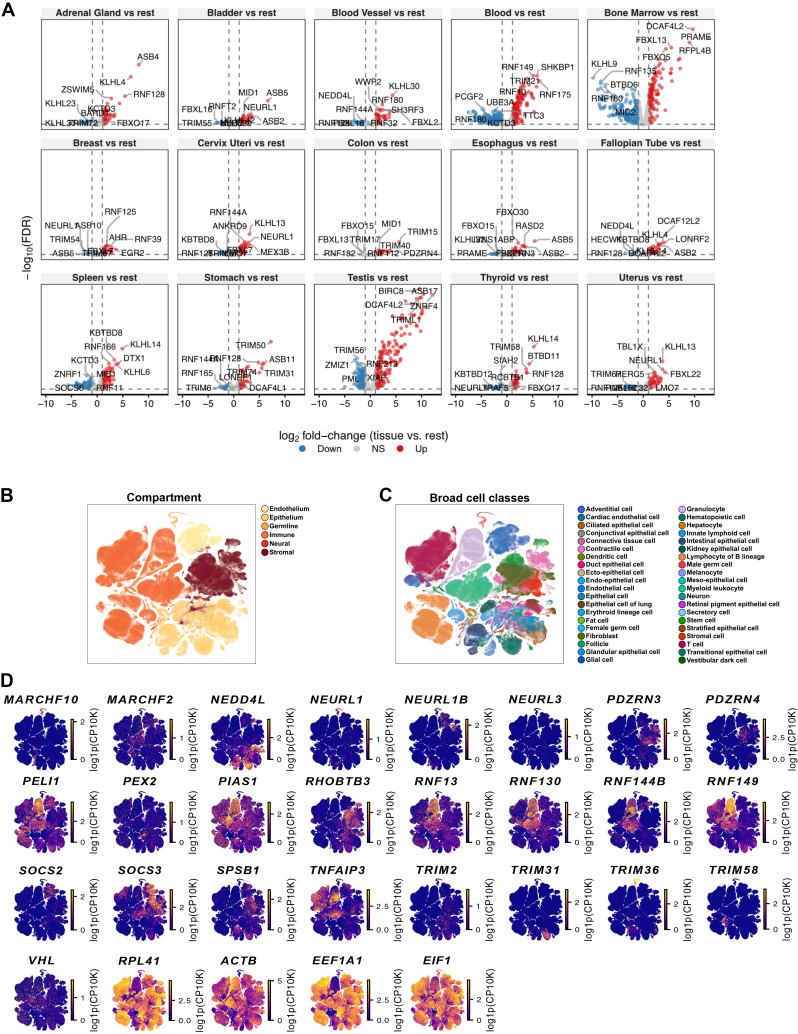
E3 expression in tissues and cells, related to Figure 6 (A) Volcano plots indicate significantly upregulated (red) or downregulated (blue) genes for each tissue compartment in a one versus all comparison. Significance was defined as FDR < 0.05 and log2 fold change ≥ 1. (B) Tabula Sapiens compartments annotation as a reference for functional compartments. (C) Tabula Sapiens broad cell class annotations as a reference for broad cell class distribution. (D) Selected E3-ome genes to illustrate compartmentalization and unique gene expression within different cell classes. *RPL41*, *ACTB*, *EEF1A1*, and *EIF1* are shown as genes expected to have broad, high cell expression.

Building on the spatial framework, we performed differential expression analyses of the human E3-ome using the GTEx dataset spanning 31 human tissues. Notably, the blood has the greatest number of differentially expressed genes (358), followed by bone marrow (295) and testis (288) (Figures 6C, 6D, and S11A; Table S1S). Genes displaying tissue-preferential upregulation included *RNF151*, *PHF7*, *FBXO47*, *TRIM42*, *TRIM60* and *RAD18* (Figure 6D). Among these, *PHF7* was significantly upregulated in the testis (Figure 6D), consistent with mouse studies showing its role in testicular hormone production.^153^^,^^154^ Similarly, the upregulated expression of *FBXO47* in the testis (Figure 6D) aligns with functional evidence that *Fbxo47*-deficient male mice are infertile.^155^ *RAD18* was preferentially upregulated in the bone marrow (Figure 6D), in which haematopoiesis takes place. Relevant to this process, it was shown that *Rad18*-deficient mice exhibit sensitivity to DNA damage in hematopoietic progenitor cells.^156^

Complementing the tissue-level data, we analyzed the Tabula Sapiens single-cell dataset to identify differentially expressed human E3-ome genes across 38 cell types (Figures 6E, 6F, and S11B–S11D; Table S1T). Among the most prominently upregulated genes were *CBLB*, *MARCHF1*, and *FBXL5*. *CBLB* was upregulated in innate lymphoid cells, fibroblasts, T cells, and stem cells (Figure 6F), consistent with CBLB roles in T cells^157^^,^^158^ and NK cells,^159^ with emerging evidence for *CBLB* specialization in fibroblasts and stem cells.^160^^,^^161^ *MARCHF1* was upregulated in lymphocytes of B lineage, myeloid leukocytes, and neurons (Figure 6F), consistent with its established roles in regulating immune receptors, influencing antigen presentation, and promoting dendritic cell maturation.^162^^,^^163^^,^^164^ Interestingly, neuron-specific upregulation suggests a role for MARCHF1 in neuronal function; however, this remains uncharacterized, whereas FBXL5 is upregulated in granulocytes, myeloid leukocytes, and kidney epithelial cells without clear evidence for specialized functions (Figure 6F). Together, these findings show that a comprehensive E3-ome enables the discovery of compelling E3s across different biological contexts.

### Structural comparisons of the RING domain reveal relationships and functional convergence

**Figure 7:**
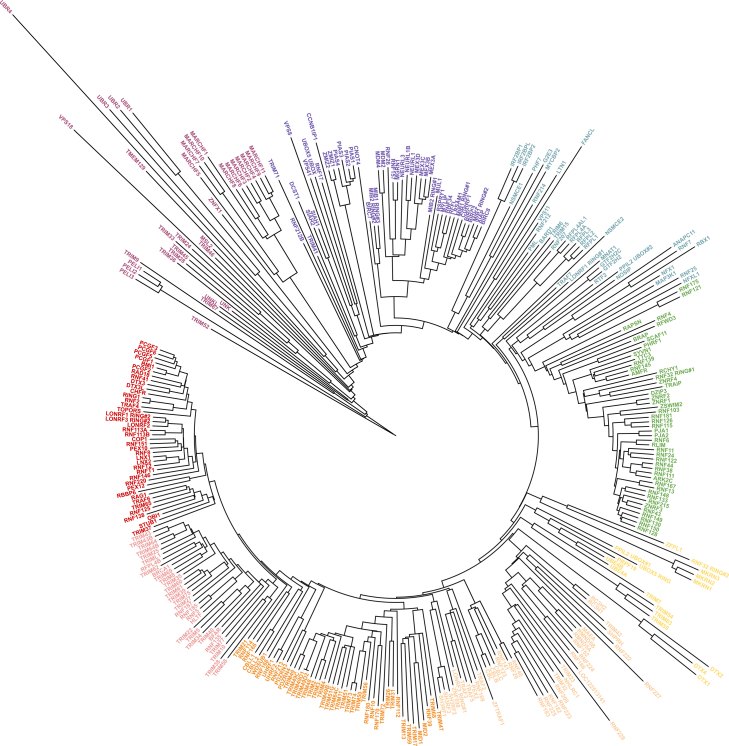
Phylogenetic tree of RING and dRING domains Dendrogram shows the similarity of the RING and dRING domains based on DALI’s all-against-all structure comparison of AlphaFold predicted structures. See also Figure S12.

**Figure S12:**
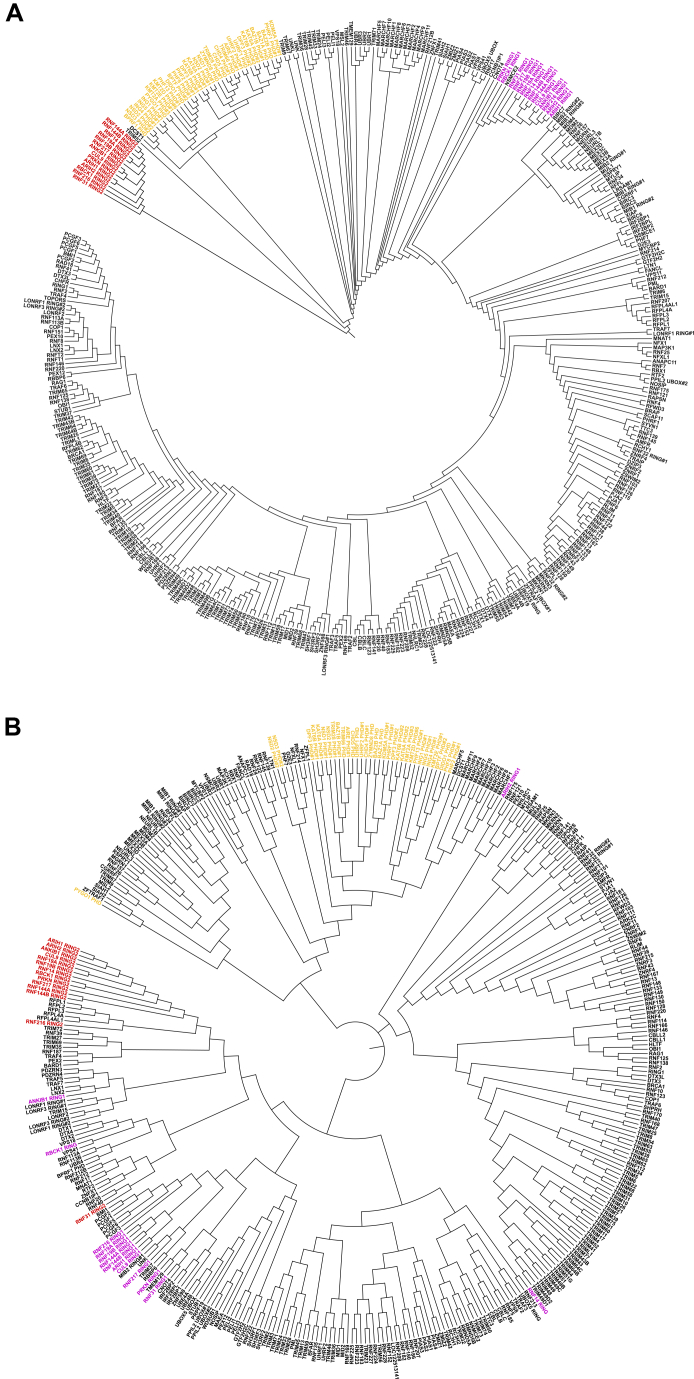
Comparison of RING, RING1, RING2, and the PHD domains, related to Figure 7 (A) Dendrogram shows structural similarity of RING, dRING, RING1 (purple), RING2 (red), and PHD (yellow) domains based on DALI’s all-against-all structure comparison. (B) Dendrogram shows sequence similarity of RING, dRING, RING1 (purple), RING2 (red), and PHD (yellow) domains based on Clustal Omega.

Although sequence-based models are tradtionally used to group E3s, this approach has limitations. Guided by Foldseek searches and the E3-ome curation, AlphaFold2 structural predictions for 309 RING, 28 dRING, 36 PHD, 14 RING1, and 14 RING2 domains were obtained (Table S1U). Using DALI’s all-against-all structural comparison, 2 dendrograms were generated (Figures 7 and S12A). The first compared RING and dRING domains (Figure 7) whereas the second compared RING, dRING, PHD, RING1, and RING2 domains (Figure S12A).

Structural comparison distinguished the PHD domains and the RING2 domain of RBRs from most RING domains effectively (Figure S12). Structural alignments of RING and dRING domains revealed functionally related clusters, including polycomb repressive complex proteins (PCGF1-6), Wnt receptor E3s (RNF43, ZNRF3), histone E3s (RNF40, RNF20), epigenetic readers (UHRF1, UHRF2), RNA binding proteins (MEX3A/3B/3C), ER quality control E3s (RNF145, RNF139, AMFR), and apoptosis-related E3s (XIAP1, MIB1, BIRC2) (Figure 7). Some of these relationships were also partially captured by sequence-based alignments (Figure S12B).

G2E3 is one instance in which a RING is mis-annotated as a PHD in databases. G2E3 contains a RING domain (residues 144–193) which, by sequence and structure, resembles the RING domain of PHF7. Although full-length G2E3 and PHF7 differ significantly in structure and sequence, they cluster within the dendrograms (Figures 7 and S12), suggesting a potential functional relationship. Supporting this, both G2E3 and PHF7 were recently identified as H3K14 E3s.^165^ This highlights how structural analysis resolves annotation conflicts and identifies functionally convergent E3s.

## Discussion

This study establishes the E3-ome, a curated census of human E3s that unifies structural, functional, and regulatory information. The E3 annotation extends beyond catalytic domain classification (e.g., RING, dRING, HECT, RBR) to a functional framework integrating biochemistry and interaction networks to define E3 families and classes. For CRLs, this expands to SR diversity by incorporating interaction data and advances in predictive resources such as AlphaFold, reflecting improved structural understanding and recently characterized SRs.

Comparisons with earlier efforts highlight the progress in our understanding of E3s. Across 11 published E3 lists and the curated human E3-ome (Figure S1B), only 242 genes are shared, representing just 36% of the 672 curated E3s defined in the E3-ome. The first curation originated in 2008,^44^ and showed an overlap of 68% with this study (517 protein-coding genes in common). The major differences come from proteins that contribute to CRL3 and CRL4 enumeration. The curation removes 37 BTB domain-containing proteins of the ZBTB series (e.g., ZBTB1, ZBTB2), 23 BTB domain-containing proteins of the KCNX (X = A / B / C / D / G, e.g., KCNA1, KCNB1, KCNC1) series, and includes 49 CRL4 SRs, some of which adopted the DCAF nomenclature (e.g., DCAF1, DCAF2), and some that do not (e.g., CRBN, ERCC8). Additional updates from this study include the identification of RING and dRING proteins (e.g., RNF227, RNF228, TMEM129, ZNFX1), SP-RING proteins (PIAS1, PIAS2, PIAS3, PIAS4), CRL1 SRs (e.g., FBXL17, FBXL19), CRL2 SRs (e.g., PRAMEF12, ZSWIM6), and atypical E3s (e.g., ZNF451, RANBP2, UBE2O, BIRC6, RNF213). These updates reflect advances in understanding and highlight the growing recognition of E3 functional and structural diversity.

E3s can be co-opted by degraders, such as molecular glues or PROTACs, and used to selectively degrade disease-related proteins.^25^ Targeted protein degradation often relies on the CRL2^VHL^ and CRL4^CRBN^ in degrader design; however, there is significant potential to broaden the range of E3s hijacked. One study carried out complementary ORFeome and arrayed screens to identify degradation or stabilization effectors.^166^ From these screens, we annotated 147 degradative effector proteins in the E3-ome, but also noted that 295 proteins in the E3-ome were not screened (Table S1A). A consistent hit protein in the screens is RNF126, an E3 involved in protein quality control,^167^^,^^168^ which has been co-opted by molecular glues.^169^ The expansion of protein effectors enhances the ability to combat a wider range of diseases more effectively, especially when degraders utilizing CRL2^VHL^ and CRL4^CRBN^ are ineffective.

We recognize the proteins within our E3-ome can function as modular systems. CRLs require additional components, such as SKP1, DDB1, ELOB, ELOC, NEDD8, and Cullin proteins to form catalytically active CRLs. Some of these core components, co-factors, and co-regulators of CRLs were present in our initial list of genes and are acknowledged (Table S1V). We also acknowledge selected complex components based on current knowledge (Tables S1V and S1W). For example, the linear Ub chain assembly complex (LUBAC) comprises RBR E3s RNF31 (also known as HOIP) and RBCK1 (also known as HOIL-1), as well as SHARPIN.^170^^,^^171^ The CTLH E3 complex includes more than just the dRING and U-box proteins (MAEA, RMND5A, RMNDB5), but also includes scaffold, SR, and supramolecular assembly proteins (e.g., RANBP9, GID8, and WDR26).^172^^,^^173^ LTN1 uses the ribosomal 60S subunit to bind several nascent polypeptides.^174^ RNF43 and ZNRF3 ubiquitinate Wnt signaling receptors, requiring the Dishevelled proteins as adaptors.^175^ There is also evidence to suggest some MAGE proteins (MAGE-A1/A2/A11/B18/C2/D1/E1/F1/G1) can interact with RING or HECT E3s to modify substrate specificity or promote E3 activity, giving rise to an unknown number of MAGE-associated-E3s.^176^^,^^177^^,^^178^^,^^179^^,^^180^^,^^181^

How else can the E3-ome landscape expand further? Though few, there are examples of E3 collaborations across E3 classes and families.^65^^,^^182^ ARIH1, ARIH2 and CUL9 are currently annotated as RBR E3s in the E3-ome, but it is also known that they can form E3 superassemblies. ARIH1 forms superassemblies with RBX1-containing neddylated CRLs *in vitro*, ARIH2 forms a superassembly with neddylated CUL5-RBX2, whereas CUL9 is a Cullin, binds RBX1, and has the RBR module in the same polypeptide.^65^^,^^135^^,^^182^ Both TRIP12, a HECT E3, and CRL2^VHL^ are also part of the E3-ome, however, what is not yet annotated is that TRIP12 and CRL2^VHL^ can form an E3-E3 complex that facilitates K29/K48-branched Ub chains on neo-substrates when utilizing CRL2^VHL^-based PROTACs.^183^ Future E3-E3 assembly studies will broaden biological insights and discover new E3 relationships within the E3-ome.

Despite our advanced understanding of the mechanisms and functions of the E3s, many E3s remain poorly defined in human physiology. Inconsistent annotations over the past 17 years have hindered unified assessments. The human E3-ome unifies E3 annotations, integrates literature, experimental data, and expert curation to standardize E3 annotation and provide a foundational resource for future research.

### Limitations of the study

We used a gene-centric approach, including genes with catalytic domains or CRL SR motifs. This generally matches experimental evidence but has exceptions, such as BRCA1-BARD1, in which only BRCA1 is catalytically active. BARD1 was nevertheless included because of its RING domain, despite the BARD1 RING lacking E2 binding and E3 activity.^38^^,^^40^ Similarly, TRAF2 was included primarily because of its RING domain, despite the domain being structurally unsuitable for E2 binding.^39^ TRAF2 and BARD1 would conventionally be labeled pseudo-E3s, akin to pseudo-kinases, and we include them as such (Table S1). Context-dependent SRs, such as CDK12, which engage CUL4 only with certain small molecules,^184^ were excluded. These examples illustrate how future updates may integrate condition-specific E3 activities. This study focused on generating the human E3-ome to reveal the membership of the human E3-ome, and while broad genetic analyses of expression and mutational status were performed using public datasets, the human E3-ome will benefit from more rigorous analyses using alternative datasets (publicly available or newly generated), using protein-centric samples, and placing the disease relevance of the human E3-ome in the context of the broader ubiquitin-protein system.

## Resource availability

### Lead contact

Requests for further information and resources should be directed to and will be fulfilled by the lead contact, Dr. Rebecca Feltham (feltham.r@wehi.edu.au).

### Materials availability

This study generated new recombinant proteins, including RNF4, RNF227, AIRE, DPF2, and TRIM29.

### Data and code availability

Data reported in this paper will be shared by the lead contact upon request. This paper does not report original code. All information about the datasets used is detailed in the method details section.

## Acknowledgments

We acknowledge the Ubiquitin Signaling Division (WEHI), Feltham Laboratory, David Barford, Satpal Virdee, Ning Zheng, Yuli Li, Christina Woo, and Cheuk Hiam Lai for assistance with curation, content reviewing, and useful scientific discussions. The laboratory of R.F. is supported by The Galbraith Family Charitable Trust, the K & M Foundation for Women, the Betty Deller King Bequest, the Berwick Opportunity Shop, Denise and Roberto Cappai, John and Tibby Peterson, and the Rae Foundation. N.K.C. is supported by a fellowship from the Marian and EH Flack Trust. S.K. is supported by NHMRC Investigator Grant (2007739) and Australian Research Council Discovery Project grants (DP240101427 and DP250100643). S.L. acknowledges support from the Max Planck Institute for Multidisciplinary Sciences, the Max Planck Society, and the SFB1565 (DFG; 469281184, P17). A.N.B. acknowledges funding from Cancer Research UK grant DRCNPG-May21∖100002 as well as from the Innovative Medicines Initiative 2 Joint Undertaking (JU) under grant agreement number 875510 (EUbOPEN). JU receives support from the European Union’s Horizon 2020 research and innovation programme, EFPIA, Ontario Institute for Cancer Research, Royal Institution for the Advancement of Learning McGill University, Kungliga Tekniska Hoegskolan, and Diamond Light Source. Research in the Ciulli laboratory on studying and targeting Cullin RING E3s has received funding from the European Research Council (ERC) under the European Union’s Seventh Framework Programme (FP7/2007-2013) as a Starting Grant to A.C. (grant agreement ERC-2012-StG-311460 DrugE3CRLs), the U.K. Biotechnology and Biological Sciences Research Council (BBSRC), and the Innovative Medicines Initiative 2 (IMI2) JU under grant agreement no 875510 (EUbOPEN project to A.C.) from the European Union’s Horizon 2020 research and innovation programme. The IMI2 JU receives support from the European Union’s Horizon 2020 research and innovation program, European Federation of Pharmaceutical Industries and Associations (EFPIA) companies, and associated partners KTH, OICR, Diamond, and McGill. The work by K.R. and J.D.-F. was funded by the Francis Crick Institute, which receives its core funding from Cancer Research United Kingdom (CC 2075), the United Kingdom Medical Research Council (CC 2075), and the Wellcome Trust (CC 2075). Work in the Hay lab was supported by an Investigator Award from Wellcome Trust (217196/Z/19/Z) and a Programme grant from Cancer Research UK (DRCRPG-May23/100003). R.P.N. is a member of the excellence cluster ImmunoSensation3 funded by the Deutsche Forschungsgemeinschaft (DFG, German Research Foundation) under Germany’s Excellence Strategy - EXC2151-390873048. This work was supported by an Australian National Health and Medical Research Council IDEAS grant (APP1136021) to J.K.P. T.J.G.-R. is an HHMI Gilliam Fellow (GT15758) and is grateful for funding from NIH Institutional training grant in cell biology (T32GM136542). M.P. is an investigator with the Howard Hughes Medical Institute, and his laboratory is supported by grant R35-GM136250 from the NIH. M.E.R. was supported by an Australian National Health and Medical Research Council (NHMRC) Investigator Grant (GNT2017257). Work by P.D.M. was funded by a Project Grant from the Health Research Council of New Zealand (18–150) and a University of Otago Postgraduate Scholarship (to C.J.R.). M.B., J.H., and J.M. sincerely thank the AstraZeneca PheWAS project teams for providing access to summary statistics and other public data resources. This work was also made possible through the Victorian State Government Operational Infrastructure Support and Australian Government National Health and Medical Research Council Independent Research Institute Infrastructure Support Scheme. M.B. was supported by an Australian National Health and Medical Research Council (NHMRC) Investigator Grant (GNT1195236). J.H. was supported by the Alfred Felton Bequest for dementia research. J.M. was supported by a CSL PhD top-up scholarship. G.G.P. is supported by a Canadian Institutes of Health Research (CIHR) Project Grant (PJT-191900). B.C.L. is supported by the National Health and Medical Research Council (NHMRC) Investigator grant GNT2016268. R.E.K. is supported by NIH grant 1 R35 GM144127. Work in the D.K. lab (WEHI) is supported by a National Health and Medical Research Council Investigator grant (GNT1178122). T.K., J.J.P., and C.D.L. were supported in part by an NIH Grant R35 GM118080 (C.D.L.). The content is solely the responsibility of the authors and does not represent the official views of the National Institutes of Health. C.D.L. is an investigator of the Howard Hughes Medical Institute. C.A.P.J. was supported by the National Institute of Neurological Disorders and Stroke (NINDS) of the NIH (R01 NS102414) and by the BMBF Cluster for the future program PROXIDRUGS, project antiMIC. J.J.B. acknowledges support from the Victorian Cancer Council. Work by K.H. was supported by the DFG (Deutsche Forschungsgemeinschaft) grant HO 3783/3-2. This work was supported by resources provided by WEHI HPC, Milton. Some schematics were generated with BioRender.com.

## Author contributions

N.K.C. and R.F. led the study, collated the E3-ome, and wrote the manuscript. N.K.C. performed sequence and structural alignments, bioinformatics analyses, and experiments and prepared all figures. Curators T.J.G.-R., C.J.R., J.D.-F., T.K., J.J.P., M.A.N., F.A., I.W., S.H., S.K., R.C.C., G.G.P., A.N.B., J.J.B., R.E.K., S.L., A.C., E.S.F., N.H.T., R.P.N., B.A.S., K.R., J.K.P., J.P.M., P.D.M., C.D.L., R.T.H., B.C.L., M.P., C.A.P.J., and K.H. extensively contributed to the E3-ome’s assembly and validation. J.M., M.B., and J.H. conducted all disease association analyses. Y.H.T. carried out experiments. M.E.R., A.S., and S.W.W. carried out bioinformatics analyses. J.I., W.A., A.G., H.H., and R.W.B. provided crucial support in data mining, analysis, and visualization. D.K., C.A.P.J., and M.R. served as advisors for the study, offering strategic guidance and expert insights.

## Declaration of interests

R.F. and B.C.L. are scientific leads at Ternarx. The Ciulli laboratory receives or has received sponsored research support from Almirall, Amgen, Amphista Therapeutics, Boehringer Ingelheim, GlaxoSmithKline, Eisai, Merck KGaA, Nurix Therapeutics, Ono Pharmaceuticals, and Tocris-Biotechne. A.C. is a co-founder and shareholder of Amphista Therapeutics and serves on the scientific advisory board (SAB) of ProtOS Bio and TrimTech therapeutics. M.P. is or has been an advisor for SEED Therapeutics, CullGen, Deargen, Kymera Therapeutics, Lumanity, Serinus Biosciences, Sibylla Biotech, Triana Biomedicines, and Umbra Therapeutics. M.P. has financial interests in CullGen, Kymera Therapeutics, SEED Therapeutics, Thermo Fisher Scientific, and Triana Biomedicines. I.W. is a co-founder and shareholder of Lyterian Therapeutics and a shareholder of Firefly Therapeutics and serves on the SAB of Firefly Therapeutics and PAIVBio. S.H. is a partner in The Column Group; a stockholder in Roche; serves on the boards of InduPro, Lyterian, Adaxion Therapeutics, and TCGFB; and is a consultant for Alector, Novartis, LTZ Therapeutics, Hexagon Bio, TCG Labs Soleil, and Surrozen. M.R. is co-founder, consultant, and SAB member to Nurix Therapeutics, Lyterian, Zenith, and Reina; SAB member of Vicinitas; and iPartner at The Column Group. P.D.M. serves in a general capacity as a SAB member for Arvinas Inc. M.B. declares her role as board director on the Australian Genome Research Facility (AGRF). D.K. is the founder, shareholder, and SAB member of Entact Bio and Proxima Bio and a co-founder of Ternarx. B.A.S. serves on the SAB of Proxygen. J.J.B. is a founder, shareholder, and SAB member of Proxima Bio. E.S.F. is a founder, SAB member, and equity holder of Civetta Therapeutics, Proximity Therapeutics, Neomorph, Inc. (also board of directors), Stelexis Biosciences, Inc., Anvia Therapeutics, Inc. (also board of directors), and CPD4, Inc. (also board of directors). He is an equity holder and SAB member for Avilar Therapeutics, Photys Therapeutics, and Ajax Therapeutics and an equity holder in Lighthorse Therapeutics. E.S.F. is a consultant to Novartis, EcoR1 Capital, Odyssey, and Deerfield. The Fischer lab receives or has received research funding from Deerfield, Novartis, Ajax, Interline, Bayer, and Astellas. C.D.L. is a co-founder and consultant to Reina Bio, Inc.

## Declaration of generative AI and AI-assisted technologies in the writing process

During the preparation of this work, the authors used ChatGPT 5.2 to improve the readability and language of the manuscript. After using this tool, the authors reviewed and edited the content as needed and take full responsibility for the content of the publication.

## STAR★Methods

### Key resources table

**Table:** 

REAGENT or RESOURCE	SOURCE	IDENTIFIER
**Antibodies**		

Anti-ubiquitin antibody	Santa Cruz Biotechnology	Cat# sc-8017: RRID: AB_62842
Peroxidase AffiniPure® Goat Anti-Mouse IgG (H+L)	Jackson ImmunoResearch	Cat# 115-035-003: RRID: AB_10015289

**Bacterial and virus strains**		

*E. coli* BL21(DE3)	Agilent Technologies	Cat# 200131

**Chemicals, peptides, recombinant proteins**		

ATP	Merck	Cat# A3377
Clarity Western ECL substrate	Bio-Rad	Cat# 1705061
Coomassie stain	Abcam	Cat# ab119211
DTT	Novachem	Cat# M02712
Glycerol	Ajax Finechem	Cat# AJA242-500ML
HEPES	Merck	Cat# H4034
IPTG	Gold Biotechnology	Cat# I2481C
Imidazole	Merck	Cat# 56749
Kanamycin	Gold Biotechnology	Cat# K-120-5
Leupeptin	Merck	Cat# L9783
Lysozyme	ChemSupply Australia	Cat# GE8228
MgCl_2_	Merck	Cat# M2670
NaCl	Ajax Finechem	Cat# AJA465-500G
Ni-NTA Reagent Kit	Merck	Cat# ACR5000NT
NuPAGE™ LDS Sample Buffer (4X)	ThermoFisher Scientific	Cat# NP0008
PMSF	Merck	Cat# P7626
Sucrose	Merck	Cat# S0389
HiLoad 16/600 Superdex 75 pg	Cytiva	Cat# 28989333
TCEP	Gold Biotechnology	Cat# TCEP25
Tris	ThermoFisher Scientific	Cat# 15504-020
Tryptone	Merck	Cat# 107213
Yeast extract	Merck	Cat# 103753
ZnCl_2_	Merck	Cat# 793523
Human His-GST-AIRE PHD (280–374)	This study	N/A
Human His-GST-AIRE PHD (293–354)	This study	N/A
Human His-GST-DPF2 PHD (270–328)	This study	N/A
Human His-GST-DPF2 PHD (270–391)	This study	N/A
Human His-GST-TRIM29 B-box (168–262)	This study	N/A
Human His-TRIM29 B-box (168–262)	This study	N/A
Human His-GST-RNF227 RING (14–119)	This study	N/A
Human RNF4 (2–190)	This study	N/A
Human UBA1	Cotton et al.^216^	N/A
Human UbcH5B	Cotton et al.^216^	N/A
Human ubiquitin	Cotton et al.^216^	N/A
Apyrase	Merck	Cat# A6237
DNase1	Merck	Cat# 04536282001
3C protease	Cotton et al.^216^	N/A

**Recombinant DNA**		

Human AIRE (280–374) gBlocks codon optimized for *E. coli* expression	Integrated DNA Technologies (IDT)	N/A
Human AIRE (293–354) gBlocks codon optimized for *E. coli* expression	Integrated DNA Technologies (IDT)	N/A
Human DPF2 (270–328) gBlocks codon optimized for *E. coli* expression	Integrated DNA Technologies (IDT)	N/A
Human DPF2 (270–391) gBlocks codon optimized for *E. coli* expression	Integrated DNA Technologies (IDT)	N/A
Human TRIM29 (168–262) gBlocks codon optimized for *E. coli* expression	Integrated DNA Technologies (IDT)	N/A
Human RNF227 (14–119) gBlocks codon optimized for *E. coli* expression	Integrated DNA Technologies (IDT)	N/A
Human RNF4 (2–190) gBlocks codon optimized for *E. coli* expression	Integrated DNA Technologies (IDT)	N/A
Plasmid: pOPINK Human AIRE (280–374)	This study	N/A
Plasmid: pOPINK Human AIRE (293–354)	This study	N/A
Plasmid: pOPINK Human DPF2 (270–328)	This study	N/A
Plasmid: pOPINK Human DPF2 (270–391)	This study	N/A
Plasmid: pOPINB Human TRIM29 (168–262)	This study	N/A
Plasmid: pOPINK HumanTRIM29 (168–262)	This study	N/A
Plasmid: pOPINK Human RNF227 (14–119)	This study	N/A
Plasmid: pOPINB Human RNF4 (2–190)	This study	N/A
Plasmid: pET-UbcH5B	Lechtenberg et al.^74^	N/A
Plasmid: pET-Ubiquitin	Lechtenberg et al.^74^	N/A
Plasmid: pOPINB	pOPINB was a gift from Ray Owens	Addgene plasmid # 41142; http://n2t.net/addgene:41142; RRID:Addgene_41142
Plasmid: pOPINK	pOPINK was a gift from Ray Owens	Addgene plasmid # 41143; http://n2t.net/addgene:41143; RRID:Addgene_41143

**Software and algorithms**		

anndata (v0.11)	Virshup et al.^217^	https://anndata.readthedocs.io/en/stable/
AlphaFold Protein Structure Database	Jumper et al. and Fleming et al.^204^^,^^213^	https://alphafold.ebi.ac.uk/
AlphaFold Server	Abramson et al.^205^	https://alphafoldserver.com/welcome
AZPheWAS Portal (470K v5)	Dhindsa et al.^185^	https://azphewas.com/
BioExcel biobb_pdb_tools	Andrio et al.^218^	https://github.com/bioexcel/biobb_pdb_tools
biomaRt (Bioconductor v3.19)	Durinck et al.^219^	https://bioconductor.org/packages/release/bioc/html/biomaRt.html
BioGRID (v4.4.233)	Oughtred et al.^209^	https://thebiogrid.org/
Clustal Omega	Madeira et al.^208^	https://www.ebi.ac.uk/jdispatcher/msa/clustalo
DaliLite v5.0.1	Holm et al.^207^	http://ekhidna2.biocenter.helsinki.fi/dali/
edgeR (v4.2.2)	Robinson et al.^199^	https://bioconductor.org/packages/release/bioc/html/edgeR.html
Ensembl (release 115)	Dyer et al.^220^	http://www.ensembl.org/
Foldseek	van Kempen et al.^221^	https://search.foldseek.com/
HGNC multi-symbol checker	HUGO Gene Nomenclature Committee	https://www.genenames.org/tools/multi-symbol-checker/
Geneious Prime® 2025.1.3	Dotmatics Geneious	https://www.geneious.com/
Genomics Data Commons Portal (release 43)	Heath et al.^222^	https://portal.gdc.cancer.gov/
GTEx (V8)	Genotype-Tissue Expression (GTEx) Portal^197^	https://gtexportal.org/home/
GWAS Catalog (v1.0.2)	Cerezo et al.^151^	https://www.ebi.ac.uk/gwas/
HPO Ontology (2025-09-01 release)	Köhler et al.^150^ Gargano et al.^210^	https://hpo.jax.org/
InterPro (release 101.0)	Blum et al.^47^	ebi.ac.uk/interpro
iTOL (v7)	Letunic et al.^223^	https://itol.embl.de/
limma (v3.60.6)	Ritchie et al.^201^	https://bioconductor.org/packages/release/bioc/html/limma.html
matplotlib (v3.10)	Hunter et al.^224^	https://matplotlib.org/
numpy (v1.26)	Harris et al.^225^	https://numpy.org/
Scanpy (v1.9.^∗^)	Wolf et al.^226^	https://scanpy.readthedocs.io/en/stable/
scipy (v1.15)	Virtanen et al.^227^	https://scipy.org/
recount3 (v1.14.0)	Wilks et al.^198^	https://rna.recount.bio/
RCSB PDB	Berman et al.^212^	https://www.rcsb.org/
Tabula Sapiens (v1 and v2)	Tabula Sapiens^202^^,^^203^	https://tabula-sapiens.sf.czbiohub.org/
UniProt	UniProt Consortium^228^	https://www.uniprot.org/
R (v4.5.2 and v4.4.2)	R foundation	https://www.r-project.org/
Fluorish	Flourish Studio	https://flourish.studio/
BioRender	BioRender	https://www.biorender.com/

**Other**		

4%–12% Bolt Bis-Tris SDS-PAGE	ThermoFisher Scientific	Cat# NW04125BOX
mPAGE®, Bis-Tris, 10x8, 4%–12%, 15 wells	Merck	Cat# MP41G15

### Method details

#### E3 classification

Sub-classified E3s from 4 prior studies^37^^,^^44^^,^^45^^,^^46^ and unpublished work from our collaborative network were collated, followed by integration and expansion using information from InterPro (release 101.0).^47^ E3s are traditionally categorized based on the type of catalytic domain and substrate receptor proteins they contain. The key catalytic domains are RING, dRINGs (e.g. SP-RING, U-box), RBR and HECT. SRs can be identified based on key protein interaction domains including F-box, BC-box-CUL2-box, BTB domain, 3-box, BC-box-CUL5-box and H-box. The presence or absence of these domains was reviewed by our curators using protein family databases, experimental structures, AlphaFold predictions, and annotations from the literature. Additionally, non-protein coding genes that were identified during our curation are listed (Table S1X).

#### Ranking of protein-coding genes

Protein-coding genes were ranked from high to low scores based on a multi-criteria evaluation system. Assessment from our curators was incorporated, where curators in the relevant protein families or classes evaluated each protein-coding gene. Curators were presented with 1 of 4 options for each gene. ‘Yes’ confirms the gene’s protein product contribution to enumeration of E3s based on supporting evidence, ‘No’ indicates the gene does not contribute to enumeration based on supporting evidence, ‘Assessed, inconclusive’ is used when the gene's protein product has been evaluated but where curators could not reach a definitive conclusion; and ‘Unassessed’ is used when the curator lacks sufficient criteria to evaluate the gene's protein product. Inclusion of a gene’s protein product for enumeration contributes an arbitrary score of 5 whilst exclusion of a gene’s protein product contributes an arbitrary score of -5. Evidence of key protein-protein interaction from BioGRID, evidence of domain presence from InterPro, evidence of domain or motif presence from literature studies were given an arbitrary score of 2 each. The sum of the scores reflects the level of confidence for inclusion of a gene’s protein product in E3 enumeration. Furthermore, genes were placed in 1 of 3 categories (category 1, category 2, or category 3). Category 1 genes are genes whereby at least one curator has included the gene’s protein product for enumeration. Category 2 genes are genes whereby no curator included the gene’s protein product for enumeration but have some evidence reported to suggest their inclusion for E3 enumeration. Category 3 genes are genes whereby no curator have included the gene’s protein product for enumeration and there is no evidence that is substantial to include the gene’s protein product for E3 enumeration.

#### AstraZeneca PheWAS portal data analyses

The AZPheWAS Portal (https://azphewas.com) is a public repository of gene-phenotype associations using data generated from the UK Biobank, (UKB, www.ukbiobank.ac.uk) consisting of over 500,000 participants, primarily UK residents aged 40–69 years. This source conducted a Phenome-wide association studies (PheWAS)^149^^,^^185^ using exome sequence data from UKB participants of European ancestry to examine the association between genes, variants, and phenotypes. We retrieved summary statistics from the publicly available AZPheWAS version 470K (v5), without accessing individual-level data. This version analyzed data from 419,391 UKB participants of European ancestry to evaluate the association between protein-coding variants and around 10,000 binary and 3,500 continuous phenotypes. These phenotypes are classified into high-level categories based on the ICD-10 classification system (https://azphewas.com/phenotypeCatalogue). We started by pre-processing the summary statistics for variant-level associations, focusing solely on significant variant-phenotype associations (p ≤ 1e-8). AZPheWAS incorporates various models for variant-level associations, including genotypic/additive, allelic, dominant, and recessive models. If a variant-phenotype association was identified across multiple models, we retained the one with the smallest p-value for further analysis. To summarize the variant-phenotype associations for E3 genes, we calculated the mean association effect for each gene across various phenotype categories. This is done by averaging the odds ratios or absolute effect sizes from all significant variant-phenotype associations within each gene, for both binary and continuous phenotypes. This reflects the estimated overall effect of each gene within each phenotype category.

#### GWAS catalog analyses

The complete GWAS Catalog association summary statistics (GWAS_Catalog_v1.0.2-associations_e114_r2025-08-24.tsv) was downloaded on 27 August 2025. The traits in the GWAS Catalog are highly diverse, encompassing a broad spectrum of diseases and phenotypes. In our analysis, we extracted key fields related to traits and genes from the 'MAPPED_TRAIT' (Mapped Experimental Factor Ontology trait) and 'MAPPED_GENE' (Gene mapped to the strongest SNP) fields, rather than the 'DISEASE/TRAIT' and 'REPORTED GENE(S)' fields reported by individual studies. Additionally, to enhance searchability, visualization, and integration of GWAS Catalog phenotype categories, we utilized the EFO Ontology Trait Mappings. (https://www.ebi.ac.uk/gwas/api/search/downloads/trait_mappings) to align the listed traits with their corresponding ontology terms and parent terms. Next, we extracted significant associations using a p-value threshold of p ≤ 5e-8 and retained only the association with the smallest p-value when the same gene-variant phenotype association was identified across multiple studies. For visualization, only variants uniquely assigned to a single gene were included and categorized as high, moderate, modifier or low impact according to Ensembl release 115^208^. We further focused on variants classified as high or moderate impact to prioritize those most likely to influence protein function. In addition, traits assigned to non-specific categories (e.g., “Other trait”, “Other disease”, “NR”, “Other measurement”) or lacking defined parent terms were excluded, as they provide limited interpretive value. In addition, the “Body measurement” category was removed because traits such as height, weight, and BMI are highly polygenic with numerous associations that may obscure more specific signals.

#### Human Phenotype Ontology (HPO) analyses

HPO version 2025-09-01 was retrieved in OBO format and the set of phenotypes associated with any of the 672 E3 genes was extracted. The gene universe was then limited to the set of 19,294 protein-coding genes recorded in the Hugo Gene Nomenclature Committee (HGNC) version 2025-09-05. Where at least 3 genes in any given E3 family or class were associated with a particular HPO term, enrichment was assessed using a one-sided Fisher’s exact test. P-values were adjusted using the Benjamini-Hochberg procedure to control false discovery rates (FDR) within each E3 family or class, and odds-ratios were calculated using the Haldane-Anscombe correction (adding a pseudocount of 0.5 to each cell). Analyses were performed using R version 4.4.2.

#### Bulk RNA-seq gene expression analyses

Bulk RNA-seq data were obtained from GTEx version V8 via recount3 (v1.14.0).^197^^,^^198^ For each tissue, one sample per donor was retained, and exactly 9 donors were randomly selected without replacement across tissues (seed = 42), ensuring that each donor contributed samples to only a single tissue. This yielded a dataset comprising 31 tissues and 279 samples. As recount3 provides coverage-level counts, matrices were converted to read-like counts by area-under-coverage (AUC) scaling to a common library size of 4×10^ˆ7^ using recount3’s transform_counts (round = FALSE), thereby placing samples on a read-count scale compatible with RNA-seq modelling. Genes with insufficient expression were removed using edgeR (v4.2.2)^199^ filterByExpr with a tissue-level design so that retention criteria reflected group structure. Library size and composition were then normalized using edgeR’s trimmed mean of M values (TMM, calcNormFactors)^200^ and the resulting effective library sizes (library size × TMM factor) were used as offsets in downstream linear models. Following this, counts data were restricted to a predefined E3 gene panel (652 genes) for further analysis. Mean variance relationships and between sample heterogeneity were modeled with limma-voom using sample quality weights (limma v3.60.6,^201^ voomWithQualityWeights) under the same tissue design. Gene-wise linear models were fit with limma’s lmFit. One-versus-rest (each tissue versus the average expression of all others) contrast was evaluated. Contrasts were applied with limma’s contrasts.fit, and statistical significance was assessed using moderated t-statistics with empirical Bayes variance moderation (eBayes, trend = TRUE, robust = TRUE). Genes were considered differentially expressed if, after Benjamini–Hochberg correction, the FDR was < 0.05 and the absolute log2 fold-change was ≥ 1. Top genes that were significantly up-regulated primarily in one tissue were selected. For visualization, each gene’s log2 fold-change values across all tissues were standardized by computing row-wise Z-scores.

#### Single cell RNA-seq gene expression analyses

E3 gene expression was analyzed using the Tabula Sapiens single-cell transcriptomic atlas.^202^^,^^203^ Raw counts were obtained from the decontXcounts layer and normalized per cell to counts per 10,000 (CP10K). Non-finite or zero entries were replaced with zeros, and library sizes were clipped to a minimum of 1 × 10⁻^12^. Cells were grouped by broad_cell_class, retaining only classes with ≥ 20 cells. Differential expression was performed in Scanpy using sc.tl.rank_genes_groups with the Wilcoxon rank-sum test in a one-versus-rest design. The sample size (n) for each comparison was the number of single cells in the focal broad_cell_class and the pooled set of all remaining cells; only classes with ≥20 cells were included. P values were adjusted for multiple testing using the Benjamini–Hochberg procedure, and statistical significance was defined as adjusted P (p_adj) < 0.05. For visualization, precomputed UMAP embeddings from the original atlas were used and each gene panel is scaled independently (vmin ≈ 0; vmax set to the gene-specific 99th percentile) to emphasise within-gene dynamic range. Gene expression was normalized per cell to CP10K and log1p-transformed for plotting. All analyses were conducted in Python 3.10 using anndata 0.11, numpy 1.26, scipy 1.15, and matplotlib 3.10.

#### Structural and sequence homology tree generation

The RING, dRING, PHD, RING1 and RING2 domains were defined and curated through manual annotation and Foldseek search, guided by existing annotations on InterPro and UniProt. Protein structure predictions were obtained from the EBI AlphaFold Protein Structure Database (https://alphafold.ebi.ac.uk/).^204^ In the case no prediction was available (proteins MYCBP2, ZFTRAF1, KMT2C, BIRC6, KMT2B, KMT2A, BPTF, KMT2D, UBR4, RNF213), AlphaFold3^205^ was used to predict the full structure. In the case of KMT2D, UBR4 and RNF213 which required too many tokens for the AlphaFold3 server (https://alphafoldserver.com/), folds were predicted by folding 2 overlapping sequences and aligning on the overlapped regions to reconstruct the entire protein prediction. The RING, dRING, PHD, RING1 and RING2 domains were extracted from the full structure using the BioExcel biobb_pdb_tools package (version 5.1.1 2025.1).^206^ The resulting PDB domains were converted to dat files using the DaliLite import.pl function and an all-against-all search performed using the -matrix option and standard parameters (DaliLite v5.0.1).^207^ The resulting Newick trees were visualized using iTOL version v7. For sequence tree generation, the sequences of the RING, dRING, PHD, RING1 and RING2 domains were aligned using Clustal Omega (https://www.ebi.ac.uk/jdispatcher/msa/clustalo)^208^ and the corresponding phylogenetics tree was visualized using iTOL version v7.

#### Databases used for analyses

InterPro (release 101.0)^47^ annotations of key domains (RING, F-box, HECT, RBR, SOCS-box) were acquired as lines of evidence of domain presence. Protein-protein interactions data was acquired from BioGRID (version 4.4.233).^209^ We annotated potential CRL SRs for reports of interactions with CUL1, CUL2, CUL3, CUL4A, CUL4B, SKP1, ELOC, ELOB, DDB1, RBX1 and RBX2. All NCBI gene IDs and gene names were cross-checked with biomaRt (Bioconductor version 3.19) and the HGNC multi-symbol checker. Gene aliases and Pfam protein domain annotation were acquired from biomaRt (Bioconductor version 3.19). For gene-phenotype associations, AZPheWAS version 470K^149^ (v5),^185^ GWAS Catalog all associations v1.0.2,^151^ HPO version 2025-09-01^210^ and HGNC version 2025-09-05^211^ were used for analyses. The Cancer Genes Census data was accessed *via* the Genomics Data Commons Portal (version 43.0). The GTEx dataset (version V8) was accessed for bulk RNA-seq analyses.^197^ The Tabula Sapiens dataset (version 1 and version 2) was used for single-cell RNA-seq analyses.^202^^,^^203^ Experimental and predicted protein structures were obtained from RSCB Protein Data Bank (RSCB PDB),^212^ AlphaFold Protein Structure Database (Google DeepMind and EMBL-EBI, https://alphafold.ebi.ac.uk)^204^ and AlphaFold Server (Google DeepMind, https://alphafoldserver.com/).^204^^,^^213^

#### Plasmids

The DNA sequences (gBlocks gene fragments, IDT Singapore) of full-length human RNF4 (amino acids 2-190), human RNF227 RING domain (amino acids 14-119), human AIRE PHD domain (amino acids 293-354 and 280-374), human DPF2 PHD domain (amino acids 270-328 and 270-391) and TRIM29 B-box (amino acids 168-262) codon-optimized for expression in *E. coli* were cloned into the pOPINB (for N-terminal 6xHis-tagged proteins) and/or pOPINK (for N-terminal 6xHis-GST-tagged proteins) vectors^214^ using in-fusion cloning (Takara Bio). The plasmids for bacterial expression of human E1, UbcH5B and ubiquitin were described previously.^74^

#### Protein expression and purification

All recombinant proteins were expressed in *E. coli* BL21(DE3). Transformed bacteria were grown at 37°C in a shaking incubator in 2xYT medium supplemented with appropriate antibiotics until OD_600_ = 0.6-0.8. The temperature was reduced to 20°C for 45 min before expression was induced with 0.5 mM IPTG. After overnight expression at 20°C, bacteria were harvested by centrifugation. Cell pellets were kept at -20°C and used for purification within 6 months. For expression of zinc-containing proteins (RNF4, RNF227, TRIM29, DPF2, AIRE), cultures were supplemented with 0.1 mM ZnCl_2_ before induction. For purification, cells were lysed by sonication in the presence of lysozyme, protease inhibitors (PMSF, leupeptin) and DNAse. Lysates were clarified by centrifugation and supernatants applied to Ni-NTA His-bind resin (from Ni-NTA Reagent Kit) equilibrated in purification buffer (50 mM Tris pH 8, 500 mM NaCl, 10% glycerol (v/v) and 10% sucrose (w/v)) to capture 6xHis-tagged proteins. Proteins were eluted in purification buffer containing 300 mM imidazole. Protein-containing fractions were pooled. RNF4 was treated with His-3C protease to remove the 6xHis-tag while dialyzing against purification buffer to remove imidazole. Cleaved RNF4 was re-applied to Ni-NTA His-bind resin to remove the His-3C protease and the cleaved His-tag and further purified *via* size-exclusion chromatography (16/600 Superdex 75 pg, Cytiva) in 20 mM HEPES pH 7.9, 150 mM NaCl, 1 mM TCEP. His- and His-GST-tagged RNF227, AIRE, DPF2 and TRIM29 were purified as described for RNF4 but without 3C protease treatment, and the size-exclusion chromatography was performed in 25 mM HEPES pH 7.9, 150 mM NaCl, 0.5 mM TCEP. UbcH5B and ubiquitin were expressed and purified as described previously.^74^ E1 was purified based on a modification of a previously published method.^215^ All proteins were aliquoted, flash frozen in liquid nitrogen and stored at -80 °C.

#### *In vitro* single-turnover E2∼Ub discharge assay

UbcH5B was charged with ubiquitin by incubating 500 nM E1, 20 μM E2, 50 μM ubiquitin, and 10 mM ATP in reaction buffer (50 mM HEPES pH 8.0, 100 mM NaCl, 10 mM MgCl_2_) at 37°C for 10 min shaking at 200 rpm. After charging is completed, apyrase is added (1 U / 100 μL). To initiate E2∼Ub discharge, 15 μL of E2∼Ub was added to 15 μL of 2 μM E3 which was prepared in reaction buffer (50 mM HEPES pH 8.0, 100 mM NaCl, 10 mM MgCl_2_). The reaction took place at room temperature, and the reaction was stopped by addition of 2X LDS sample. Samples were placed on ice immediately, resolved by 4-12% Bolt Bis-Tris SDS-PAGE gel or 4-12% mPAGE® Bis-Tris followed by Coomassie stain.

#### *In vitro* auto-ubiquitination assay

Ubiquitination chain formation assays were carried out by mixing 0.1 μM E1, 1 μM E2, 1 μM E3 and 20 μM Ub. The reaction was carried out in 50 mM HEPES pH 8.0, 100 mM NaCl, 0.6 mM DTT, 10 mM MgCl_2_ and 10 mM ATP in 50 μL reaction volume. Samples were taken at 2 h and 3 h, terminated by adding samples to 2X LDS-sample buffer with 100 mM DTT. The samples were run on reducing 4-12% Bolt Bis-Tris SDS-PAGE gels and transferred onto 0.2 μm PVDF membrane. The membranes were detected with ubiquitin antibody (P4D1) and Clarity™ Western ECL Substrate.

### Quantification and statistical analysis

All statistical analysis and software used are described within the Method details section.

## Supplemental information

Table S1. E3-ome curation, related to Figures 1–7 and S1–S12

## References

[1] Scheffner M., Nuber U., Huibregtse J.M. (1995). Protein ubiquitination involving an E1-E2-E3 enzyme ubiquitin thioester cascade. Nature.

[2] Hershko A., Ciechanover A. (1998). The ubiquitin system. Annu. Rev. Biochem..

[3] Cappadocia L., Lima C.D. (2018). Ubiquitin-like Protein Conjugation: Structures, Chemistry, and Mechanism. Chem. Rev..

[4] Buetow L., Huang D.T. (2016). Structural insights into the catalysis and regulation of E3 ubiquitin ligases. Nat. Rev. Mol. Cell Biol..

[5] Metzger M.B., Hristova V.A., Weissman A.M. (2012). HECT and RING finger families of E3 ubiquitin ligases at a glance. J. Cell Sci..

[6] Morreale F.E., Walden H. (2016). Types of Ubiquitin Ligases. Cell.

[7] Berndsen C.E., Wolberger C. (2014). New insights into ubiquitin E3 ligase mechanism. Nat. Struct. Mol. Biol..

[8] Dikic I., Schulman B.A. (2023). An expanded lexicon for the ubiquitin code. Nat. Rev. Mol. Cell Biol..

[9] Hu H., Sun S.C. (2016). Ubiquitin signaling in immune responses. Cell Res..

[10] Chauhan A.S., Jhujh S.S., Stewart G.S. (2024). E3 ligases: a ubiquitous link between DNA repair, DNA replication and human disease. Biochem. J..

[11] Padovani C., Jevtić P., Rapé M. (2022). Quality control of protein complex composition. Mol. Cell.

[12] Kandel R., Jung J., Neal S. (2024). Proteotoxic stress and the ubiquitin proteasome system. Semin. Cell Dev. Biol..

[13] Kevei É., Pokrzywa W., Hoppe T. (2017). Repair or destruction-an intimate liaison between ubiquitin ligases and molecular chaperones in proteostasis. FEBS Lett..

[14] Lechtenberg B.C., Komander D. (2024). Just how big is the ubiquitin system?. Nat. Struct. Mol. Biol..

[15] Otten E.G., Werner E., Crespillo-Casado A., Boyle K.B., Dharamdasani V., Pathe C., Santhanam B., Randow F. (2021). Ubiquitylation of lipopolysaccharide by RNF213 during bacterial infection. Nature.

[16] Aravind L., Koonin E.V. (2000). The U box is a modified RING finger - a common domain in ubiquitination. Curr. Biol..

[17] Hochstrasser M. (2001). SP-RING for SUMO: new functions bloom for a ubiquitin-like protein. Cell.

[18] Harper J.W., Schulman B.A. (2021). Cullin-RING Ubiquitin Ligase Regulatory Circuits: A Quarter Century Beyond the F-Box Hypothesis. Annu. Rev. Biochem..

[19] Hopf L.V.M., Baek K., Klügel M., von Gronau S., Xiong Y., Schulman B.A. (2022). Structure of CRL7(FBXW8) reveals coupling with CUL1-RBX1/ROC1 for multi-cullin-RING E3-catalyzed ubiquitin ligation. Nat. Struct. Mol. Biol..

[20] Lücking C.B., Dürr A., Bonifati V., Vaughan J., De Michele G., Gasser T., Harhangi B.S., Meco G., Denèfle P., Wood N.W. (2000). Association between early-onset Parkinson's disease and mutations in the parkin gene. N. Engl. J. Med..

[21] Onel K., Cordon-Cardo C. (2004). MDM2 and prognosis. Mol. Cancer Res..

[22] Oliner J.D., Saiki A.Y., Caenepeel S. (2016). The Role of MDM2 Amplification and Overexpression in Tumorigenesis. Cold Spring Harb. Perspect. Med..

[23] Wäsch R., Engelbert D. (2005). Anaphase-promoting complex-dependent proteolysis of cell cycle regulators and genomic instability of cancer cells. Oncogene.

[24] Sansregret L., Swanton C. (2017). The Role of Aneuploidy in Cancer Evolution. Cold Spring Harb. Perspect. Med..

[25] Békés M., Langley D.R., Crews C.M. (2022). PROTAC targeted protein degraders: the past is prologue. Nat. Rev. Drug Discov..

[26] Jevtić P., Haakonsen D.L., Rapé M. (2021). An E3 ligase guide to the galaxy of small-molecule-induced protein degradation. Cell Chem. Biol..

[27] Duan S., Pagano M. (2021). Ubiquitin ligases in cancer: Functions and clinical potentials. Cell Chem. Biol..

[28] Wertz I.E., Wang X. (2019). From Discovery to Bedside: Targeting the Ubiquitin System. Cell Chem. Biol..

[29] Zhao X. (2018). SUMO-Mediated Regulation of Nuclear Functions and Signaling Processes. Mol. Cell.

[30] Rabut G., Peter M. (2008). Function and regulation of protein neddylation. ‘Protein modifications: beyond the usual suspects’ review series. EMBO Rep..

[31] Schmidtke G., Aichem A., Groettrup M. (2014). FAT10ylation as a signal for proteasomal degradation. Biochim. Biophys. Acta.

[32] Zhou X., Mahdizadeh S.J., Le Gallo M., Eriksson L.A., Chevet E., Lafont E. (2024). UFMylation: a ubiquitin-like modification. Trends Biochem. Sci..

[33] Perng Y.C., Lenschow D.J. (2018). ISG15 in antiviral immunity and beyond. Nat. Rev. Microbiol..

[34] Li Z., Chen S., Jhong J.H., Pang Y., Huang K.Y., Li S., Lee T.Y. (2021).

[35] Liu L., Damerell D.R., Koukouflis L., Tong Y., Marsden B.D., Schapira M. (2019). UbiHub: a data hub for the explorers of ubiquitination pathways. Bioinformatics.

[36] Liu Y., Yang J., Wang T., Luo M., Chen Y., Chen C., Ronai Z., Zhou Y., Ruppin E., Han L. (2023). Expanding PROTACtable genome universe of E3 ligases. Nat. Commun..

[37] Medvar B., Raghuram V., Pisitkun T., Sarkar A., Knepper M.A. (2016). Comprehensive database of human E3 ubiquitin ligases: application to aquaporin-2 regulation. Physiol. Genomics.

[38] Brzovic P.S., Keeffe J.R., Nishikawa H., Miyamoto K., Fox D., Fukuda M., Ohta T., Klevit R. (2003). Binding and recognition in the assembly of an active BRCA1/BARD1 ubiquitin-ligase complex. Proc. Natl. Acad. Sci. USA.

[39] Yin Q., Lamothe B., Darnay B.G., Wu H. (2009). Structural basis for the lack of E2 interaction in the RING domain of TRAF2. Biochemistry.

[40] Christensen D.E., Brzovic P.S., Klevit R.E. (2007). E2-BRCA1 RING interactions dictate synthesis of mono- or specific polyubiquitin chain linkages. Nat. Struct. Mol. Biol..

[41] Buchwald G., van der Stoop P., Weichenrieder O., Perrakis A., van Lohuizen M., Sixma T.K. (2006). Structure and E3-ligase activity of the Ring-Ring complex of polycomb proteins Bmi1 and Ring1b. EMBO J..

[42] Reverter D., Lima C.D. (2005). Insights into E3 ligase activity revealed by a SUMO-RanGAP1-Ubc9-Nup358 complex. Nature.

[43] Pichler A., Gast A., Seeler J.S., Dejean A., Melchior F. (2002). The nucleoporin RanBP2 has SUMO1 E3 ligase activity. Cell.

[44] Li W., Bengtson M.H., Ulbrich A., Matsuda A., Reddy V.A., Orth A., Chanda S.K., Batalov S., Joazeiro C.A.P. (2008). Genome-wide and functional annotation of human E3 ubiquitin ligases identifies MULAN, a mitochondrial E3 that regulates the organelle's dynamics and signaling. PLOS One.

[45] Ge Z., Leighton J.S., Wang Y., Peng X., Chen Z., Chen H., Sun Y., Yao F., Li J., Zhang H. (2018). Integrated Genomic Analysis of the Ubiquitin Pathway across Cancer Types. Cell Rep..

[46] Hundley F.V., Sanvisens Delgado N., Marin H.C., Carr K.L., Tian R., Toczyski D.P. (2021). A comprehensive phenotypic CRISPR-Cas9 screen of the ubiquitin pathway uncovers roles of ubiquitin ligases in mitosis. Mol. Cell.

[47] Blum M., Andreeva A., Florentino L.C., Chuguransky S.R., Grego T., Hobbs E., Pinto B.L., Orr A., Paysan-Lafosse T., Ponamareva I. (2025). InterPro: the protein sequence classification resource in 2025. Nucleic Acids Res..

[48] Vittal V., Stewart M.D., Brzovic P.S., Klevit R.E. (2015). Regulating the Regulators: Recent Revelations in the Control of E3 Ubiquitin Ligases. J. Biol. Chem..

[49] Deshaies R.J., Joazeiro C.A.P. (2009). RING domain E3 ubiquitin ligases. Annu. Rev. Biochem..

[50] Freemont P.S., Hanson I.M., Trowsdale J. (1991). A novel cysteine-rich sequence motif. Cell.

[51] Zheng N., Wang P., Jeffrey P.D., Pavletich N.P. (2000). Structure of a c-Cbl-UbcH7 complex: RING domain function in ubiquitin-protein ligases. Cell.

[52] Mace P.D., Linke K., Feltham R., Schumacher F.R., Smith C.A., Vaux D.L., Silke J., Day C.L. (2008). Structures of the cIAP2 RING domain reveal conformational changes associated with ubiquitin-conjugating enzyme (E2) recruitment. J. Biol. Chem..

[53] Yin Q., Lin S.C., Lamothe B., Lu M., Lo Y.C., Hura G., Zheng L., Rich R.L., Campos A.D., Myszka D.G. (2009). E2 interaction and dimerization in the crystal structure of TRAF6. Nat. Struct. Mol. Biol..

[54] Plechanovová A., Jaffray E.G., McMahon S.A., Johnson K.A., Navrátilová I., Naismith J.H., Hay R.T. (2011). Mechanism of ubiquitylation by dimeric RING ligase RNF4. Nat. Struct. Mol. Biol..

[55] Sampson D.A., Wang M., Matunis M.J. (2001). The small ubiquitin-like modifier-1 (SUMO-1) consensus sequence mediates Ubc9 binding and is essential for SUMO-1 modification. J. Biol. Chem..

[56] Tatham M.H., Jaffray E., Vaughan O.A., Desterro J.M., Botting C.H., Naismith J.H., Hay R.T. (2001). Polymeric chains of SUMO-2 and SUMO-3 are conjugated to protein substrates by SAE1/SAE2 and Ubc9. J. Biol. Chem..

[57] Capili A.D., Lima C.D. (2007). Structure and analysis of a complex between SUMO and Ubc9 illustrates features of a conserved E2-Ubl interaction. J. Mol. Biol..

[58] Streich F.C., Lima C.D. (2016). Capturing a substrate in an activated RING E3/E2-SUMO complex. Nature.

[59] Vander Kooi C.W., Ohi M.D., Rosenberg J.A., Oldham M.L., Newcomer M.E., Gould K.L., Chazin W.J. (2006). The Prp19 U-box crystal structure suggests a common dimeric architecture for a class of oligomeric E3 ubiquitin ligases. Biochemistry.

[60] Hatakeyama S., Yada M., Matsumoto M., Ishida N., Nakayama K.I. (2001). U box proteins as a new family of ubiquitin-protein ligases. J. Biol. Chem..

[61] Zhang M., Windheim M., Roe S.M., Peggie M., Cohen P., Prodromou C., Pearl L.H. (2005). Chaperoned ubiquitylation--crystal structures of the CHIP U box E3 ubiquitin ligase and a CHIP-Ubc13-Uev1a complex. Mol. Cell.

[62] Barnsby-Greer L., Mabbitt P.D., Dery M.A., Squair D.R., Wood N.T., Lamoliatte F., Lange S.M., Virdee S. (2024). UBE2A and UBE2B are recruited by an atypical E3 ligase module in UBR4. Nat. Struct. Mol. Biol..

[63] Zheng N., Schulman B.A., Song L., Miller J.J., Jeffrey P.D., Wang P., Chu C., Koepp D.M., Elledge S.J., Pagano M. (2002). Structure of the Cul1-Rbx1-Skp1-F boxSkp2 SCF ubiquitin ligase complex. Nature.

[64] Höfler A., Yu J., Yang J., Zhang Z., Chang L., McLaughlin S.H., Grime G.W., Garman E.F., Boland A., Barford D. (2024). Cryo-EM structures of apo-APC/C and APC/CCDH1:EMI1 complexes provide insights into APC/C regulation. Nat. Commun..

[65] Kostrhon S., Prabu J.R., Baek K., Horn-Ghetko D., von Gronau S., Klügel M., Basquin J., Alpi A.F., Schulman B.A. (2021). CUL5-ARIH2 E3-E3 ubiquitin ligase structure reveals cullin-specific NEDD8 activation. Nat. Chem. Biol..

[66] Rotin D., Kumar S. (2009). Physiological functions of the HECT family of ubiquitin ligases. Nat. Rev. Mol. Cell Biol..

[67] Huang L., Kinnucan E., Wang G., Beaudenon S., Howley P.M., Huibregtse J.M., Pavletich N.P. (1999). Structure of an E6AP-UbcH7 complex: insights into ubiquitination by the E2-E3 enzyme cascade. Science.

[68] Lorenz S. (2018). Structural mechanisms of HECT-type ubiquitin ligases. Biol. Chem..

[69] Huibregtse J.M., Scheffner M., Beaudenon S., Howley P.M. (1995). A family of proteins structurally and functionally related to the E6-AP ubiquitin-protein ligase. Proc. Natl. Acad. Sci. USA.

[70] Wenzel D.M., Lissounov A., Brzovic P.S., Klevit R.E. (2011). UBCH7 reactivity profile reveals parkin and HHARI to be RING/HECT hybrids. Nature.

[71] Spratt D.E., Walden H., Shaw G.S. (2014). RBR E3 ubiquitin ligases: new structures, new insights, new questions. Biochem. J..

[72] Dove K.K., Stieglitz B., Duncan E.D., Rittinger K., Klevit R.E. (2016). Molecular insights into RBR E3 ligase ubiquitin transfer mechanisms. EMBO Rep..

[73] Cotton T.R., Lechtenberg B.C. (2020). Chain reactions: molecular mechanisms of RBR ubiquitin ligases. Biochem. Soc. Trans..

[74] Lechtenberg B.C., Rajput A., Sanishvili R., Dobaczewska M.K., Ware C.F., Mace P.D., Riedl S.J. (2016). Structure of a HOIP/E2∼ubiquitin complex reveals RBR E3 ligase mechanism and regulation. Nature.

[75] Pao K.C., Wood N.T., Knebel A., Rafie K., Stanley M., Mabbitt P.D., Sundaramoorthy R., Hofmann K., van Aalten D.M.F., Virdee S. (2018). Activity-based E3 ligase profiling uncovers an E3 ligase with esterification activity. Nature.

[76] Mabbitt P.D., Loreto A., Déry M.A., Fletcher A.J., Stanley M., Pao K.C., Wood N.T., Coleman M.P., Virdee S. (2020). Structural basis for RING-Cys-Relay E3 ligase activity and its role in axon integrity. Nat. Chem. Biol..

[77] Eisenhardt N., Chaugule V.K., Koidl S., Droescher M., Dogan E., Rettich J., Sutinen P., Imanishi S.Y., Hofmann K., Palvimo J.J. (2015). A new vertebrate SUMO enzyme family reveals insights into SUMO-chain assembly. Nat. Struct. Mol. Biol..

[78] Cappadocia L., Pichler A., Lima C.D. (2015). Structural basis for catalytic activation by the human ZNF451 SUMO E3 ligase. Nat. Struct. Mol. Biol..

[79] Metlagel Z., Otomo C., Takaesu G., Otomo T. (2013). Structural basis of ATG3 recognition by the autophagic ubiquitin-like protein ATG12. Proc. Natl. Acad. Sci. USA.

[80] Hanada T., Noda N.N., Satomi Y., Ichimura Y., Fujioka Y., Takao T., Inagaki F., Ohsumi Y. (2007). The Atg12-Atg5 conjugate has a novel E3-like activity for protein lipidation in autophagy. J. Biol. Chem..

[81] Otomo C., Metlagel Z., Takaesu G., Otomo T. (2013). Structure of the human ATG12∼ATG5 conjugate required for LC3 lipidation in autophagy. Nat. Struct. Mol. Biol..

[82] Tatsumi K., Sou Y.S., Tada N., Nakamura E., Iemura S., Natsume T., Kang S.H., Chung C.H., Kasahara M., Kominami E. (2010). A novel type of E3 ligase for the Ufm1 conjugation system. J. Biol. Chem..

[83] Ishimura R., Ito S., Mao G., Komatsu-Hirota S., Inada T., Noda N.N., Komatsu M. (2023). Mechanistic insights into the roles of the UFM1 E3 ligase complex in ufmylation and ribosome-associated protein quality control. Sci. Adv..

[84] Peter J.J., Magnussen H.M., DaRosa P.A., Millrine D., Matthews S.P., Lamoliatte F., Sundaramoorthy R., Kopito R.R., Kulathu Y. (2022). A non-canonical scaffold-type E3 ligase complex mediates protein UFMylation. EMBO J..

[85] Yanagitani K., Juszkiewicz S., Hegde R.S. (2017). UBE2O is a quality control factor for orphans of multiprotein complexes. Science.

[86] Nguyen A.T., Prado M.A., Schmidt P.J., Sendamarai A.K., Wilson-Grady J.T., Min M., Campagna D.R., Tian G., Shi Y., Dederer V. (2017). UBE2O remodels the proteome during terminal erythroid differentiation. Science.

[87] Yip M.C.J., Sedor S.F., Shao S. (2022). Mechanism of client selection by the protein quality-control factor UBE2O. Nat. Struct. Mol. Biol..

[88] Ehrmann J.F., Grabarczyk D.B., Heinke M., Deszcz L., Kurzbauer R., Hudecz O., Shulkina A., Gogova R., Meinhart A., Versteeg G.A. (2023). Structural basis for regulation of apoptosis and autophagy by the BIRC6/SMAC complex. Science.

[89] Mashtalir N., Daou S., Barbour H., Sen N.N., Gagnon J., Hammond-Martel I., Dar H.H., Therrien M., Affar el B. (2014). Autodeubiquitination protects the tumor suppressor BAP1 from cytoplasmic sequestration mediated by the atypical ubiquitin ligase UBE2O. Mol. Cell.

[90] Chitalia V.C., Foy R.L., Bachschmid M.M., Zeng L., Panchenko M.V., Zhou M.I., Bharti A., Seldin D.C., Lecker S.H., Dominguez I. (2008). Jade-1 inhibits Wnt signalling by ubiquitylating beta-catenin and mediates Wnt pathway inhibition by pVHL. Nat. Cell Biol..

[91] Han X., Gui B., Xiong C., Zhao L., Liang J., Sun L., Yang X., Yu W., Si W., Yan R. (2014). Destabilizing LSD1 by Jade-2 promotes neurogenesis: an antibraking system in neural development. Mol. Cell.

[92] Wang J., Muntean A.G., Wu L., Hess J.L. (2012). A subset of mixed lineage leukemia proteins has plant homeodomain (PHD)-mediated E3 ligase activity. J. Biol. Chem..

[93] Liu C., Zhang D., Shen Y., Tao X., Liu L., Zhong Y., Fang S. (2015). DPF2 regulates OCT4 protein level and nuclear distribution. Biochim. Biophys. Acta.

[94] Xiong X., Panchenko T., Yang S., Zhao S., Yan P., Zhang W., Xie W., Li Y., Zhao Y., Allis C.D. (2016). Selective recognition of histone crotonylation by double PHD fingers of MOZ and DPF2. Nat. Chem. Biol..

[95] Sanchez R., Zhou M.M. (2011). The PHD finger: a versatile epigenome reader. Trends Biochem. Sci..

[96] Jain K., Fraser C.S., Marunde M.R., Parker M.M., Sagum C., Burg J.M., Hall N., Popova I.K., Rodriguez K.L., Vaidya A. (2020). Characterization of the plant homeodomain (PHD) reader family for their histone tail interactions. Epigenetics Chromatin.

[97] Uchida D., Hatakeyama S., Matsushima A., Han H., Ishido S., Hotta H., Kudoh J., Shimizu N., Doucas V., Nakayama K.I. (2004). AIRE functions as an E3 ubiquitin ligase. J. Exp. Med..

[98] Bottomley M.J., Stier G., Pennacchini D., Legube G., Simon B., Akhtar A., Sattler M., Musco G. (2005). NMR structure of the first PHD finger of autoimmune regulator protein (AIRE1). Insights into autoimmune polyendocrinopathy-candidiasis-ectodermal dystrophy (APECED) disease. J. Biol. Chem..

[99] Lee H.S., Bang I., You J., Jeong T.K., Kim C.R., Hwang M., Kim J.S., Baek S.H., Song J.J., Choi H.J. (2023). Molecular basis for PHF7-mediated ubiquitination of histone H3. Genes Dev..

[100] Lu Z., Xu S., Joazeiro C., Cobb M.H., Hunter T. (2002). The PHD domain of MEKK1 acts as an E3 ubiquitin ligase and mediates ubiquitination and degradation of ERK1/2. Mol. Cell.

[101] Aravind L., Iyer L.M., Koonin E.V. (2003). Scores of RINGS but no PHDs in ubiquitin signaling. Cell Cycle.

[102] Scheel H., Hofmann K. (2003). No evidence for PHD fingers as ubiquitin ligases. Trends Cell Biol..

[103] Boamah D., Lin T., Poppinga F.A., Basu S., Rahman S., Essel F., Chakravarty S. (2018). Characteristics of a PHD Finger Subtype. Biochemistry.

[104] Slama P., Geman D. (2011). Identification of family-determining residues in PHD fingers. Nucleic Acids Res..

[105] Bienz M. (2006). The PHD finger, a nuclear protein-interaction domain. Trends Biochem. Sci..

[106] Liu Y., Tempel W., Zhang Q., Liang X., Loppnau P., Qin S., Min J. (2016). Family-wide Characterization of Histone Binding Abilities of Human CW Domain-containing Proteins. J. Biol. Chem..

[107] Albanese K.I., Waters M.L. (2021). Contributions of methionine to recognition of trimethyllysine in aromatic cage of PHD domains: implications of polarizability, hydrophobicity, and charge on binding. Chem. Sci..

[108] Jin X., Jin H.R., Jung H.S., Lee S.J., Lee J.H., Lee J.J. (2010). An atypical E3 ligase zinc finger protein 91 stabilizes and activates NF-kappaB-inducing kinase via Lys63-linked ubiquitination. J. Biol. Chem..

[109] Bell J.L., Malyukova A., Holien J.K., Koach J., Parker M.W., Kavallaris M., Marshall G.M., Cheung B.B. (2012). TRIM16 acts as an E3 ubiquitin ligase and can heterodimerize with other TRIM family members. PLOS One.

[110] Han X., Du H., Massiah M.A. (2011). Detection and characterization of the in vitro e3 ligase activity of the human MID1 protein. J. Mol. Biol..

[111] Lee S., Tsai Y.C., Mattera R., Smith W.J., Kostelansky M.S., Weissman A.M., Bonifacino J.S., Hurley J.H. (2006). Structural basis for ubiquitin recognition and autoubiquitination by Rabex-5. Nat. Struct. Mol. Biol..

[112] Mattera R., Tsai Y.C., Weissman A.M., Bonifacino J.S. (2006). The Rab5 guanine nucleotide exchange factor Rabex-5 binds ubiquitin (Ub) and functions as a Ub ligase through an atypical Ub-interacting motif and a zinc finger domain. J. Biol. Chem..

[113] Komander D., Lord C.J., Scheel H., Swift S., Hofmann K., Ashworth A., Barford D. (2008). The structure of the CYLD USP domain explains its specificity for Lys63-linked polyubiquitin and reveals a B box module. Mol. Cell.

[114] Mahrour N., Redwine W.B., Florens L., Swanson S.K., Martin-Brown S., Bradford W.D., Staehling-Hampton K., Washburn M.P., Conaway R.C., Conaway J.W. (2008). Characterization of Cullin-box sequences that direct recruitment of Cul2-Rbx1 and Cul5-Rbx2 modules to Elongin BC-based ubiquitin ligases. J. Biol. Chem..

[115] Okumura F., Fujiki Y., Oki N., Osaki K., Nishikimi A., Fukui Y., Nakatsukasa K., Kamura T. (2020). Cul5-type Ubiquitin Ligase KLHDC1 Contributes to the Elimination of Truncated SELENOS Produced by Failed UGA/Sec Decoding. iScience.

[116] Okumura F., Matsuzaki M., Nakatsukasa K., Kamura T. (2012). The Role of Elongin BC-Containing Ubiquitin Ligases. Front. Oncol..

[117] Krek W. (2003). BTB proteins as henchmen of Cul3-based ubiquitin ligases. Nat. Cell Biol..

[118] Canning P., Cooper C.D.O., Krojer T., Murray J.W., Pike A.C.W., Chaikuad A., Keates T., Thangaratnarajah C., Hojzan V., Marsden B.D. (2013). Structural basis for Cul3 protein assembly with the BTB-Kelch family of E3 ubiquitin ligases. J. Biol. Chem..

[119] Pintard L., Willems A., Peter M. (2004). Cullin-based ubiquitin ligases: Cul3-BTB complexes join the family. EMBO J..

[120] Zhuang M., Calabrese M.F., Liu J., Waddell M.B., Nourse A., Hammel M., Miller D.J., Walden H., Duda D.M., Seyedin S.N. (2009). Structures of SPOP-substrate complexes: insights into molecular architectures of BTB-Cul3 ubiquitin ligases. Mol. Cell.

[121] Errington W.J., Khan M.Q., Bueler S.A., Rubinstein J.L., Chakrabartty A., Privé G.G. (2012). Adaptor protein self-assembly drives the control of a cullin-RING ubiquitin ligase. Structure.

[122] Stogios P.J., Downs G.S., Jauhal J.J.S., Nandra S.K., Privé G.G. (2005). Sequence and structural analysis of BTB domain proteins. Genome Biol..

[123] Jiang W., Wang W., Kong Y., Zheng S. (2023). Structural basis for the ubiquitination of G protein βγ subunits by KCTD5/Cullin3 E3 ligase. Sci. Adv..

[124] Pinkas D.M., Sanvitale C.E., Bufton J.C., Sorrell F.J., Solcan N., Chalk R., Doutch J., Bullock A.N. (2017). Structural complexity in the KCTD family of Cullin3-dependent E3 ubiquitin ligases. Biochem. J..

[125] Balasco N., Esposito L., Smaldone G., Salvatore M., Vitagliano L. (2024). A Comprehensive Analysis of the Structural Recognition between KCTD Proteins and Cullin 3. Int. J. Mol. Sci..

[126] Nguyen D.M., Rath D.H., Devost D., Pétrin D., Rizk R., Ji A.X., Narayanan N., Yong D., Zhai A., Kuntz D.A. (2024). Structure and dynamics of a pentameric KCTD5/CUL3/Gβγ E3 ubiquitin ligase complex. Proc. Natl. Acad. Sci. USA.

[127] Scrima A., Fischer E.S., Lingaraju G.M., Böhm K., Cavadini S., Thomä N.H. (2011). Detecting UV-lesions in the genome: The modular CRL4 ubiquitin ligase does it best!. FEBS Lett..

[128] Fischer E.S., Scrima A., Böhm K., Matsumoto S., Lingaraju G.M., Faty M., Yasuda T., Cavadini S., Wakasugi M., Hanaoka F. (2011). The molecular basis of CRL4DDB2/CSA ubiquitin ligase architecture, targeting, and activation. Cell.

[129] Li T., Robert E.I., van Breugel P.C., Strubin M., Zheng N. (2010). A promiscuous alpha-helical motif anchors viral hijackers and substrate receptors to the CUL4-DDB1 ubiquitin ligase machinery. Nat. Struct. Mol. Biol..

[130] Mohamed W.I., Schenk A.D., Kempf G., Cavadini S., Basters A., Potenza A., Abdul Rahman W., Rabl J., Reichermeier K., Thomä N.H. (2021). The CRL4(DCAF1) cullin-RING ubiquitin ligase is activated following a switch in oligomerization state. EMBO J..

[131] Scrima A., Konícková R., Czyzewski B.K., Kawasaki Y., Jeffrey P.D., Groisman R., Nakatani Y., Iwai S., Pavletich N.P., Thomä N.H. (2008). Structural basis of UV DNA-damage recognition by the DDB1-DDB2 complex. Cell.

[132] Fischer E.S., Böhm K., Lydeard J.R., Yang H., Stadler M.B., Cavadini S., Nagel J., Serluca F., Acker V., Lingaraju G.M. (2014). Structure of the DDB1-CRBN E3 ubiquitin ligase in complex with thalidomide. Nature.

[133] Kramer E.R., Gieffers C., Hölzl G., Hengstschläger M., Peters J.M. (1998). Activation of the human anaphase-promoting complex by proteins of the CDC20/Fizzy family. Curr. Biol..

[134] Thornton B.R., Ng T.M., Matyskiela M.E., Carroll C.W., Morgan D.O., Toczyski D.P. (2006). An architectural map of the anaphase-promoting complex. Genes Dev..

[135] Horn-Ghetko D., Hopf L.V.M., Tripathi-Giesgen I., Du J., Kostrhon S., Vu D.T., Beier V., Steigenberger B., Prabu J.R., Stier L. (2024). Noncanonical assembly, neddylation and chimeric cullin-RING/RBR ubiquitylation by the 1.8 MDa CUL9 E3 ligase complex. Nat. Struct. Mol. Biol..

[136] Scott D.C., Dharuman S., Griffith E., Chai S.C., Ronnebaum J., King M.T., Tangallapally R., Lee C., Gee C.T., Yang L. (2024). Principles of paralog-specific targeted protein degradation engaging the C-degron E3 KLHDC2. Nat. Commun..

[137] Grabarczyk D.B., Aird E.J., Reznikow V., Kirchgatterer P.C., Ehrmann J.F., Kurzbauer R., Bell L.E., Kellner M.J., Aggarwal R., Schleiffer A. (2025). A split-site E3 ligase mechanism enables ZNFX1 to ubiquitinate and cluster single-stranded RNA into ubiquitin-coated nucleoprotein particles. Cell.

[138] Paluda A., Middleton A.J., Rossig C., Mace P.D., Day C.L. (2022). Ubiquitin and a charged loop regulate the ubiquitin E3 ligase activity of Ark2C. Nat. Commun..

[139] Maspero E., Valentini E., Mari S., Cecatiello V., Soffientini P., Pasqualato S., Polo S. (2013). Structure of a ubiquitin-loaded HECT ligase reveals the molecular basis for catalytic priming. Nat. Struct. Mol. Biol..

[140] Fletcher A.J., Mallery D.L., Watkinson R.E., Dickson C.F., James L.C. (2015). Sequential ubiquitination and deubiquitination enzymes synchronize the dual sensor and effector functions of TRIM21. Proc. Natl. Acad. Sci. USA.

[141] Lee D.F., Kuo H.P., Liu M., Chou C.K., Xia W., Du Y., Shen J., Chen C.T., Huo L., Hsu M.C. (2009). KEAP1 E3 ligase-mediated downregulation of NF-kappaB signaling by targeting IKKbeta. Mol. Cell.

[142] Jeong Y., Hellyer J.A., Stehr H., Hoang N.T., Niu X., Das M., Padda S.K., Ramchandran K., Neal J.W., Wakelee H. (2020). Role of KEAP1/NFE2L2 Mutations in the Chemotherapeutic Response of Patients with Non-Small Cell Lung Cancer. Clin. Cancer Res..

[143] Padmanabhan B., Tong K.I., Ohta T., Nakamura Y., Scharlock M., Ohtsuji M., Kang M.I., Kobayashi A., Yokoyama S., Yamamoto M. (2006). Structural basis for defects of Keap1 activity provoked by its point mutations in lung cancer. Mol. Cell.

[144] Rechsteiner M.P., von Teichman A., Nowicka A., Sulser T., Schraml P., Moch H. (2011). VHL gene mutations and their effects on hypoxia inducible factor HIFα: identification of potential driver and passenger mutations. Cancer Res..

[145] Forman J.R., Worth C.L., Bickerton G.R.J., Eisen T.G., Blundell T.L. (2009). Structural bioinformatics mutation analysis reveals genotype-phenotype correlations in von Hippel-Lindau disease and suggests molecular mechanisms of tumorigenesis. Proteins.

[146] Guey S., Kraemer M., Hervé D., Ludwig T., Kossorotoff M., Bergametti F., Schwitalla J.C., Choi S., Broseus L., Callebaut I. (2017). Rare RNF213 variants in the C-terminal region encompassing the RING-finger domain are associated with moyamoya angiopathy in Caucasians. Eur. J. Hum. Genet..

[147] Takeda M., Tezuka T., Kim M., Choi J., Oichi Y., Kobayashi H., Harada K.H., Mizushima T., Taketani S., Koizumi A. (2020). Moyamoya disease patient mutations in the RING domain of RNF213 reduce its ubiquitin ligase activity and enhance NFκB activation and apoptosis in an AAA+ domain-dependent manner. Biochem. Biophys. Res. Commun..

[148] Ahel J., Lehner A., Vogel A., Schleiffer A., Meinhart A., Haselbach D., Clausen T. (2020). Moyamoya disease factor RNF213 is a giant E3 ligase with a dynein-like core and a distinct ubiquitin-transfer mechanism. eLife.

[149] Wang Q., Dhindsa R.S., Carss K., Harper A.R., Nag A., Tachmazidou I., Vitsios D., Deevi S.V.V., Mackay A., Muthas D. (2021). Rare variant contribution to human disease in 281,104 UK Biobank exomes. Nature.

[150] Köhler S., Gargano M., Matentzoglu N., Carmody L.C., Lewis-Smith D., Vasilevsky N.A., Danis D., Balagura G., Baynam G., Brower A.M. (2021). The Human Phenotype Ontology in 2021. Nucleic Acids Res..

[151] Cerezo M., Sollis E., Ji Y., Lewis E., Abid A., Bircan K.O., Hall P., Hayhurst J., John S., Mosaku A. (2025). The NHGRI-EBI GWAS Catalog: standards for reusability, sustainability and diversity. Nucleic Acids Res..

[152] Hein M.Y., Peng D., Todorova V., McCarthy F., Kim K., Liu C., Savy L., Januel C., Baltazar-Nunez R., Sekhar M. (2025). Global organelle profiling reveals subcellular localization and remodeling at proteome scale. Cell.

[153] Kim J.E., Park S.G., Ka D.B., Kim E.K., Cho S.M., Kim H.R., Lee M.N., Choi K.C., Yoon W.K., Nam K.H. (2024). Phf7 has impacts on the body growth and bone remodeling by regulating testicular hormones in male mice. Biochem. Biophys. Res. Commun..

[154] Kim C.R., Noda T., Kim H., Kim G., Park S., Na Y., Oura S., Shimada K., Bang I., Ahn J.Y. (2020). PHF7 Modulates BRDT Stability and Histone-to-Protamine Exchange during Spermiogenesis. Cell Rep..

[155] Hua R., Wei H., Liu C., Zhang Y., Liu S., Guo Y., Cui Y., Zhang X., Guo X., Li W. (2019). FBXO47 regulates telomere-inner nuclear envelope integration by stabilizing TRF2 during meiosis. Nucleic Acids Res..

[156] Yang Y., Poe J.C., Yang L., Fedoriw A., Desai S., Magnuson T., Li Z., Fedoriw Y., Araki K., Gao Y. (2016). Rad18 confers hematopoietic progenitor cell DNA damage tolerance independently of the Fanconi Anemia pathway in vivo. Nucleic Acids Res..

[157] Eggert J., Zinzow-Kramer W.M., Hu Y., Kolawole E.M., Tsai Y.L., Weiss A., Evavold B.D., Salaita K., Scharer C.D., Au-Yeung B.B. (2024). Cbl-b mitigates the responsiveness of naive CD8(+) T cells that experience extensive tonic T cell receptor signaling. Sci. Signal..

[158] Bachmaier K., Krawczyk C., Kozieradzki I., Kong Y.Y., Sasaki T., Oliveira-Dos-Santos A., Mariathasan S., Bouchard D., Wakeham A., Itie A. (2000). Negative regulation of lymphocyte activation and autoimmunity by the molecular adaptor Cbl-b. Nature.

[159] Paolino M., Choidas A., Wallner S., Pranjic B., Uribesalgo I., Loeser S., Jamieson A.M., Langdon W.Y., Ikeda F., Fededa J.P. (2014). The E3 ligase Cbl-b and TAM receptors regulate cancer metastasis via natural killer cells. Nature.

[160] Zutshi N., Mohapatra B.C., Mondal P., An W., Goetz B.T., Wang S., Li S., Storck M.D., Mercer D.F., Black A.R. (2024). Cbl and Cbl-b ubiquitin ligases are essential for intestinal epithelial stem cell maintenance. iScience.

[161] Rorsman C., Tsioumpekou M., Heldin C.H., Lennartsson J. (2016). The Ubiquitin Ligases c-Cbl and Cbl-b Negatively Regulate Platelet-derived Growth Factor (PDGF) BB-induced Chemotaxis by Affecting PDGF Receptor β (PDGFRβ) Internalization and Signaling. J. Biol. Chem..

[162] Matsuki Y., Ohmura-Hoshino M., Goto E., Aoki M., Mito-Yoshida M., Uematsu M., Hasegawa T., Koseki H., Ohara O., Nakayama M. (2007). Novel regulation of MHC class II function in B cells. EMBO J..

[163] Ohmura-Hoshino M., Matsuki Y., Mito-Yoshida M., Goto E., Aoki-Kawasumi M., Nakayama M., Ohara O., Ishido S. (2009). Cutting edge: requirement of MARCH-I-mediated MHC II ubiquitination for the maintenance of conventional dendritic cells. J. Immunol..

[164] De Gassart A., Camosseto V., Thibodeau J., Ceppi M., Catalan N., Pierre P., Gatti E. (2008). MHC class II stabilization at the surface of human dendritic cells is the result of maturation-dependent MARCH I down-regulation. Proc. Natl. Acad. Sci. USA.

[165] Huang Y., Sun Y., Qi H., Jiang Q., Li J., Chang M., Li X., Shu L., Duan X., Wang Y. (2025). A conserved H3K14ub-driven H3K9me3 for chromatin compartmentalization. Nature.

[166] Poirson J., Cho H., Dhillon A., Haider S., Imrit A.Z., Lam M.H.Y., Alerasool N., Lacoste J., Mizan L., Wong C. (2024). Proteome-scale discovery of protein degradation and stabilization effectors. Nature.

[167] Rodrigo-Brenni M.C., Gutierrez E., Hegde R.S. (2014). Cytosolic quality control of mislocalized proteins requires RNF126 recruitment to Bag6. Mol. Cell.

[168] Hu X., Wang L., Wang Y., Ji J., Li J., Wang Z., Li C., Zhang Y., Zhang Z.R. (2020). RNF126-Mediated Reubiquitination Is Required for Proteasomal Degradation of p97-Extracted Membrane Proteins. Mol. Cell.

[169] Toriki E.S., Papatzimas J.W., Nishikawa K., Dovala D., Frank A.O., Hesse M.J., Dankova D., Song J.G., Bruce-Smythe M., Struble H. (2023). Rational Chemical Design of Molecular Glue Degraders. ACS Cent. Sci..

[170] Ikeda F., Deribe Y.L., Skånland S.S., Stieglitz B., Grabbe C., Franz-Wachtel M., van Wijk S.J.L., Goswami P., Nagy V., Terzic J. (2011). SHARPIN forms a linear ubiquitin ligase complex regulating NF-κB activity and apoptosis. Nature.

[171] Gerlach B., Cordier S.M., Schmukle A.C., Emmerich C.H., Rieser E., Haas T.L., Webb A.I., Rickard J.A., Anderton H., Wong W.W.L. (2011). Linear ubiquitination prevents inflammation and regulates immune signalling. Nature.

[172] Gottemukkala K.V., Chrustowicz J., Sherpa D., Sepic S., Vu D.T., Karayel Ö., Papadopoulou E.C., Gross A., Schorpp K., von Gronau S. (2024). Non-canonical substrate recognition by the human WDR26-CTLH E3 ligase regulates prodrug metabolism. Mol. Cell.

[173] Maitland M.E.R., Onea G., Chiasson C.A., Wang X., Ma J., Moor S.E., Barber K.R., Lajoie G.A., Shaw G.S., Schild-Poulter C. (2019). The mammalian CTLH complex is an E3 ubiquitin ligase that targets its subunit muskelin for degradation. Sci. Rep..

[174] Joazeiro C.A.P. (2019). Mechanisms and functions of ribosome-associated protein quality control. Nat. Rev. Mol. Cell Biol..

[175] Jiang X., Charlat O., Zamponi R., Yang Y., Cong F. (2015). Dishevelled promotes Wnt receptor degradation through recruitment of ZNRF3/RNF43 E3 ubiquitin ligases. Mol. Cell.

[176] Yang S.W., Huang X., Lin W., Min J., Miller D.J., Mayasundari A., Rodrigues P., Griffith E.C., Gee C.T., Li L. (2020). Structural basis for substrate recognition and chemical inhibition of oncogenic MAGE ubiquitin ligases. Nat. Commun..

[177] Pineda C.T., Ramanathan S., Fon Tacer K., Weon J.L., Potts M.B., Ou Y.H., White M.A., Potts P.R. (2015). Degradation of AMPK by a cancer-specific ubiquitin ligase. Cell.

[178] Yang S.W., Li L., Connelly J.P., Porter S.N., Kodali K., Gan H., Park J.M., Tacer K.F., Tillman H., Peng J. (2020). A Cancer-Specific Ubiquitin Ligase Drives mRNA Alternative Polyadenylation by Ubiquitinating the mRNA 3′ End Processing Complex. Mol. Cell.

[179] Griffith-Jones S., Álvarez L., Mukhopadhyay U., Gharbi S., Rettel M., Adams M., Hennig J., Bhogaraju S. (2024). Structural basis for RAD18 regulation by MAGEA4 and its implications for RING ubiquitin ligase binding by MAGE family proteins. EMBO J..

[180] Weon J.L., Yang S.W., Potts P.R. (2018). Cytosolic Iron-Sulfur Assembly Is Evolutionarily Tuned by a Cancer-Amplified Ubiquitin Ligase. Mol. Cell.

[181] Kozakova L., Vondrova L., Stejskal K., Charalabous P., Kolesar P., Lehmann A.R., Uldrijan S., Sanderson C.M., Zdrahal Z., Palecek J.J. (2015). The melanoma-associated antigen 1 (MAGEA1) protein stimulates the E3 ubiquitin-ligase activity of TRIM31 within a TRIM31-MAGEA1-NSE4 complex. Cell Cycle.

[182] Horn-Ghetko D., Krist D.T., Prabu J.R., Baek K., Mulder M.P.C., Klügel M., Scott D.C., Ovaa H., Kleiger G., Schulman B.A. (2021). Ubiquitin ligation to F-box protein targets by SCF-RBR E3-E3 super-assembly. Nature.

[183] Kaiho-Soma A., Akizuki Y., Igarashi K., Endo A., Shoda T., Kawase Y., Demizu Y., Naito M., Saeki Y., Tanaka K. (2021). TRIP12 promotes small-molecule-induced degradation through K29/K48-branched ubiquitin chains. Mol. Cell.

[184] Ghosh P., Schmitz M., Pandurangan T., Zeleke S.T., Chan S.C., Mosior J., Sun L., Palve V., Grassie D., Anand K. (2024). Discovery and design of molecular glue enhancers of CDK12-DDB1 interactions for targeted degradation of cyclin K. RSC Chem. Biol..

[185] Dhindsa R.S., Burren O.S., Sun B.B., Prins B.P., Matelska D., Wheeler E., Mitchell J., Oerton E., Hristova V.A., Smith K.R. (2023). Rare variant associations with plasma protein levels in the UK Biobank. Nature.

[186] Krissinel E., Henrick K. (2007). Inference of macromolecular assemblies from crystalline state. J. Mol. Biol..

[187] Wu G., Xu G., Schulman B.A., Jeffrey P.D., Harper J.W., Pavletich N.P. (2003). Structure of a beta-TrCP1-Skp1-beta-catenin complex: destruction motif binding and lysine specificity of the SCF(beta-TrCP1) ubiquitin ligase. Mol. Cell.

[188] Nguyen H.C., Yang H., Fribourgh J.L., Wolfe L.S., Xiong Y. (2015). Insights into Cullin-RING E3 ubiquitin ligase recruitment: structure of the VHL-EloBC-Cul2 complex. Structure.

[189] Stebbins C.E., Kaelin W.G., Pavletich N.P. (1999). Structure of the VHL-ElonginC-ElonginB complex: implications for VHL tumor suppressor function. Science.

[190] Cardote T.A.F., Gadd M.S., Ciulli A. (2017). Crystal Structure of the Cul2-Rbx1-EloBC-VHL Ubiquitin Ligase Complex. Structure.

[191] Bullock A.N., Debreczeni J.E., Edwards A.M., Sundström M., Knapp S. (2006). Crystal structure of the SOCS2-elongin C-elongin B complex defines a prototypical SOCS box ubiquitin ligase. Proc. Natl. Acad. Sci. USA.

[192] Kim Y.K., Kwak M.J., Ku B., Suh H.Y., Joo K., Lee J., Jung J.U., Oh B.H. (2013). Structural basis of intersubunit recognition in elongin BC-cullin 5-SOCS box ubiquitin-protein ligase complexes. Acta Crystallogr., D.

[193] Babon J.J., Sabo J.K., Zhang J.G., Nicola N.A., Norton R.S. (2009). The SOCS box encodes a hierarchy of affinities for Cullin5: implications for ubiquitin ligase formation and cytokine signalling suppression. J. Mol. Biol..

[194] Kamura T., Maenaka K., Kotoshiba S., Matsumoto M., Kohda D., Conaway R.C., Conaway J.W., Nakayama K.I. (2004). VHL-box and SOCS-box domains determine binding specificity for Cul2-Rbx1 and Cul5-Rbx2 modules of ubiquitin ligases. Genes Dev..

[195] Duncan E.D., Lencer E., Linklater E., Prekeris R. (2021). Methods to Study the Unique SOCS Box Domain of the Rab40 Small GTPase Subfamily. Methods Mol. Biol..

[196] Ji A.X., Privé G.G. (2013). Crystal structure of KLHL3 in complex with Cullin3. PLOS One.

[197] GTEx Consortium (2020). The GTEx Consortium atlas of genetic regulatory effects across human tissues. Science.

[198] Wilks C., Zheng S.C., Chen F.Y., Charles R., Solomon B., Ling J.P., Imada E.L., Zhang D., Joseph L., Leek J.T. (2021). recount3: summaries and queries for large-scale RNA-seq expression and splicing. Genome Biol..

[199] Robinson M.D., McCarthy D.J., Smyth G.K. (2010). edgeR: a Bioconductor package for differential expression analysis of digital gene expression data. Bioinformatics.

[200] Robinson M.D., Oshlack A. (2010). A scaling normalization method for differential expression analysis of RNA-seq data. Genome Biol..

[201] Ritchie M.E., Phipson B., Wu D., Hu Y., Law C.W., Shi W., Smyth G.K. (2015). limma powers differential expression analyses for RNA-sequencing and microarray studies. Nucleic Acids Res..

[202] Jones R.C., Karkanias J., Krasnow M.A., Pisco A.O., Quake S.R., Salzman J., Yosef N., Bulthaup B., Brown P., et al., Tabula Sapiens Consortium^∗^ (2022). The Tabula Sapiens: A multiple-organ, single-cell transcriptomic atlas of humans. Science.

[203] Quake S.R., Tabula Sapiens Consortium (2025). Tabula Sapiens reveals transcription factor expression, senescence effects, and sex-specific features in cell types from 28 human organs and tissues. bioRxiv.

[204] Fleming J., Magana P., Nair S., Tsenkov M., Bertoni D., Pidruchna I., Lima Afonso M.Q., Midlik A., Paramval U., Žídek A. (2025). AlphaFold Protein Structure Database and 3D-Beacons: New Data and Capabilities. J. Mol. Biol..

[205] Abramson J., Adler J., Dunger J., Evans R., Green T., Pritzel A., Ronneberger O., Willmore L., Ballard A.J., Bambrick J. (2024). Accurate structure prediction of biomolecular interactions with AlphaFold 3. Nature.

[206] Rodrigues J.P.G.L.M., Teixeira J.M.C., Trellet M., Bonvin A.M.J.J. (2018). pdb-tools: a swiss army knife for molecular structures. F1000Res.

[207] Holm L. (2019). Benchmarking fold detection by DaliLite v.5. Bioinformatics.

[208] Madeira F., Madhusoodanan N., Lee J., Eusebi A., Niewielska A., Tivey A.R.N., Lopez R., Butcher S. (2024). The EMBL-EBI Job Dispatcher sequence analysis tools framework in 2024. Nucleic Acids Res..

[209] Oughtred R., Rust J., Chang C., Breitkreutz B.J., Stark C., Willems A., Boucher L., Leung G., Kolas N., Zhang F. (2021). The BioGRID database: A comprehensive biomedical resource of curated protein, genetic, and chemical interactions. Protein Sci..

[210] Gargano M.A., Matentzoglu N., Coleman B., Addo-Lartey E.B., Anagnostopoulos A.V., Anderton J., Avillach P., Bagley A.M., Bakštein E., Balhoff J.P. (2024). The Human Phenotype Ontology in 2024: phenotypes around the world. Nucleic Acids Res..

[211] Tweedie S., Braschi B., Gray K., Jones T.E.M., Seal R.L., Yates B., Bruford E.A. (2021). Genenames.org: the HGNC and VGNC resources in 2021. Nucleic Acids Res..

[212] Berman H.M., Westbrook J., Feng Z., Gilliland G., Bhat T.N., Weissig H., Shindyalov I.N., Bourne P.E. (2000). The Protein Data Bank. Nucleic Acids Res..

[213] Jumper J., Evans R., Pritzel A., Green T., Figurnov M., Ronneberger O., Tunyasuvunakool K., Bates R., Žídek A., Potapenko A. (2021). Highly accurate protein structure prediction with AlphaFold. Nature.

[214] Berrow N.S., Alderton D., Sainsbury S., Nettleship J., Assenberg R., Rahman N., Stuart D.I., Owens R.J. (2007). A versatile ligation-independent cloning method suitable for high-throughput expression screening applications. Nucleic Acids Res..

[215] Tongaonkar P., Madura K. (1998). Reconstituting ubiquitination reactions with affinity-purified components and 32P-ubiquitin. Anal. Biochem..

[216] Cotton T.R., Cobbold S.A., Bernardini J.P., Richardson L.W., Wang X.S., Lechtenberg B.C. (2022). Structural basis of K63-ubiquitin chain formation by the Gordon-Holmes syndrome RBR E3 ubiquitin ligase RNF216. Mol. Cell.

[217] Virshup I., Rybakov S., Theis F.J., Angerer P., Wolf F.A. (2024). anndata: Access and store annotated data matrices. J. Open Source Softw..

[218] Andrio P., Hospital A., Conejero J., Jordá L., Del Pino M., Codo L., Soiland-Reyes S., Goble C., Lezzi D., Badia R.M. (2019). BioExcel Building Blocks, a software library for interoperable biomolecular simulation workflows. Sci. Data.

[219] Durinck S., Spellman P.T., Birney E., Huber W. (2009). Mapping identifiers for the integration of genomic datasets with the R/Bioconductor package biomaRt. Nat. Protoc..

[220] Dyer S.C., Austine-Orimoloye O., Azov A.G., Barba M., Barnes I., Barrera-Enriquez V.P., Becker A., Bennett R., Beracochea M., Berry A. (2025). Ensembl 2025. Nucleic Acids Res..

[221] van Kempen M., Kim S.S., Tumescheit C., Mirdita M., Lee J., Gilchrist C.L.M., Söding J., Steinegger M. (2024). Fast and accurate protein structure search with Foldseek. Nat. Biotechnol..

[222] Heath A.P., Ferretti V., Agrawal S., An M., Angelakos J.C., Arya R., Bajari R., Baqar B., Barnowski J.H.B., Burt J. (2021). The NCI Genomic Data Commons. Nat. Genet..

[223] Letunic I., Bork P. (2024). Interactive Tree of Life (iTOL) v6: recent updates to the phylogenetic tree display and annotation tool. Nucleic Acids Res..

[224] Hunter J.D. (2007). Matplotlib: A 2D Graphics Environment. Comput. Sci. Eng..

[225] Harris C.R., Millman K.J., van der Walt S.J., Gommers R., Virtanen P., Cournapeau D., Wieser E., Taylor J., Berg S., Smith N.J. (2020). Array programming with NumPy. Nature.

[226] Wolf F.A., Angerer P., Theis F.J. (2018). SCANPY: large-scale single-cell gene expression data analysis. Genome Biol..

[227] Virtanen P., Gommers R., Oliphant T.E., Haberland M., Reddy T., Cournapeau D., Burovski E., Peterson P., Weckesser W., Bright J. (2020). SciPy 1.0: fundamental algorithms for scientific computing in Python. Nat. Methods.

[228] UniProt Consortium (2025). UniProt: the Universal Protein Knowledgebase in 2025. Nucleic Acids Res..

